# A synthesis of the external morphology of cypridiform larvae of Facetotecta (crustacea: Thecostraca) and the limits of the genus *Hansenocaris*

**DOI:** 10.1002/ece3.9488

**Published:** 2022-11-17

**Authors:** Gregory A. Kolbasov, Alexandra S. Savchenko, Niklas Dreyer, Benny K. K. Chan, Jens T. Høeg

**Affiliations:** ^1^ White Sea Biological Station Biological Faculty of Moscow State University Moscow Russia; ^2^ Invertebrate Zoology Department, Biological Faculty Moscow State University Moscow Russia; ^3^ Biodiversity Research Center Academia Sinica Taipei Taiwan; ^4^ Natural History Museum of Denmark University of Copenhagen Copenhagen Denmark; ^5^ Department of Life Science National Taiwan Normal University Taipei Taiwan; ^6^ Biodiversity Program, Taiwan International Graduate Program Academia Sinica Taipei Taiwan; ^7^ Marine Biology Section, Department of Biology University of Copenhagen Copenhagen Denmark

**Keywords:** Facetotecta, morphology, phylogeny, taxonomy, ultrastructure, Y‐cyprids

## Abstract

Although the naupliar and cypridiform stages of the enigmatic y‐larvae of Facetotecta have been found in the marine plankton worldwide, they still represent the last significant group of crustaceans for which the adult forms are still unknown. From a number of y‐cyprids representing different taxa from different locations, we employ scanning electron microscopy to describe fine morphological details of all external structures of this unique larval form. We document different segmentation patterns of the abdomen and presence/absence of the labrum and structural differences in the antennules, labrum, paraocular process, thoracopods, and telson lend support for the erection of several new genera as opposed to the single *Hansenocaris*. The data presented here emphasize the morphological limits of the genus *Hansenocaris* and the “bauplan” of cyprydiform larvae of Facetotecta. Although the optimum pathway is a joint analysis of both molecular and morphological characters, we use the morphological characters of y‐cyprids to align them cladistically and determine the limits of the genus *Hansenocaris* s.s. and describe common characters for all y‐cyprids including six pairs of the lattice organs instead five pairs considered as a ground pattern for all Thecostraca. We also determine plesiomorphic and apomorphic characters of all known y‐cyprids and separate them from other thecostracan cypridiform larvae.

## Graphical abstract

Facetotecta is also named as Y larvae which is an extreme poorly known subclass in Thecostraca. At present, only the parasitic larvae was discovered in the marine environment but not the adults and its hosts. The present MS documents different morphological parts of Facetotecta and lends support for the erection of several new genera as opposed to the single genus Hansenocaris in Facetotecta. We use the morphological characters of y‐cyprids to align them cladistically and determine the limits of the genus *Hansenocaris* s.s., and describe common characters for all y‐cyprids including six pairs of the lattice organs instead five pairs considered as a ground pattern for all Thecostraca. We also determine plesiomorphic and apomorphic characters of all known y‐cyprids, and separate them from other thecostracan cypridiform larvae.

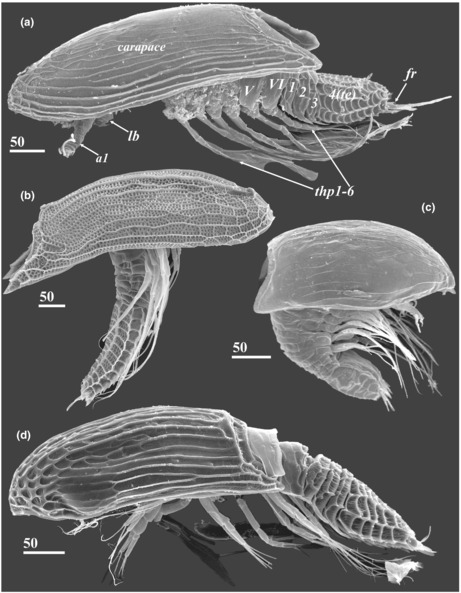

## 1 INTRODUCTION

**FIGURE 1:**
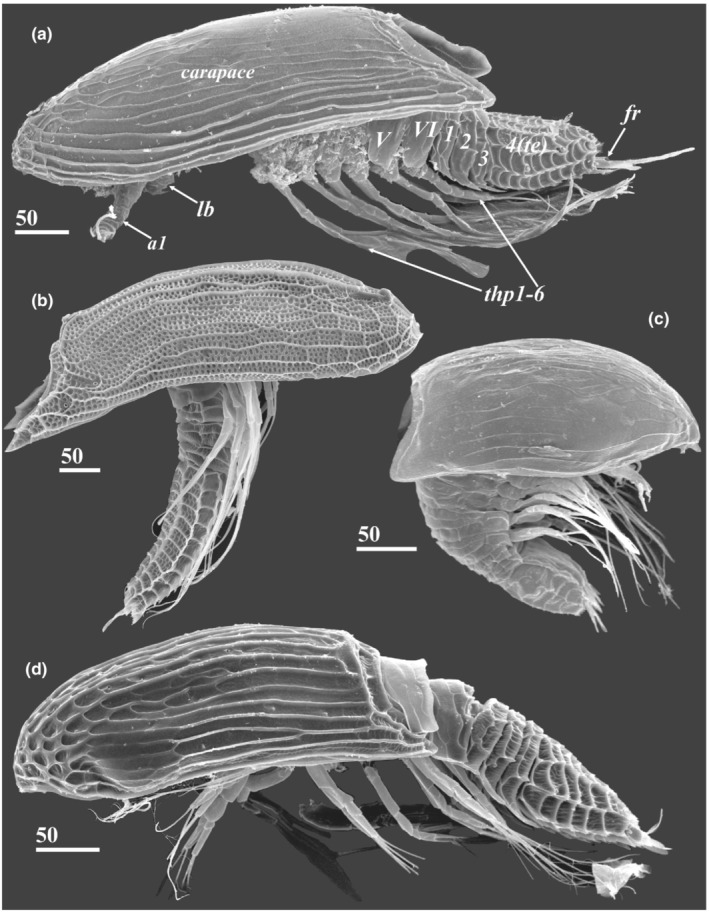
Diversity of y‐cypris larvae of Facetotecta, general view, lateral side (SEM). (a) *H. itoi* from arctic White Sea, subtidal zone, thoracic segments numbered in Roman, abdominal segments numbered in Arabic. (b) Y‐cypris larva from boreal Kuril‐Kamchatka trench, abyssal zone, depth 3000–5640 m. (c) *H. papillata* from equatorial Indonesia, subtidal zone. (d) *H. spiridonovi* from subtropical Azores Islands, subtidal zone. *a1*—antennule; *fr*—furcal rami; *lb*—labrum; *te*—telson; *thp1‐6*—thoracopods 1–6. Scale bars in μm.

Although the naupliar and cypridiform stages of the enigmatic y‐larvae of Facetotecta have been found in the marine plankton worldwide, they still represent the last significant group of crustaceans for which the adult forms are still unknown (Glenner et al., 2008; Grygier, 1996; Høeg et al., 2014; Kolbasov et al., 2021a; Kolbasov & Høeg, 2003). Different facetotectan nauplii were first described in detail more than 100 years ago by Hansen (1899), who originally illustrated five different naupliar types of y‐larvae from West Indian, equatorial Atlantic waters, and from the Bay of Kiel in the Baltic. Subsequently, y‐larvae were reported from almost all oceans in the world (Belmonte, 2005; Kolbasov et al., 2021a; Kolbasov & Høeg, 2003; Ponomarenko & Korn, 2006). A post‐naupliar instar or “y‐cyprid” (Figure 1), resembling other thecostracan cypridiform larvae, was first described by Bresciani (1965). Treatment with the crustacean molting hormone 20‐hydroxy ecdysone has been shown to induce y‐cyprids to molt into a unique minute, slug‐like stage, called the ypsigon (Glenner et al., 2008). The morphology of both the y‐cyprid and the ypsigon suggest that unknown adult stages are advanced endoparasites in still to be identified hosts (Glenner et al., 2008; Pérez‐Losada et al., 2009). Thus, the incompletely known life cycle of Facetotecta includes free‐swimming naupliar stages, a cypridiform larva specialized for attachment and an ypsigon with an unknown role (Høeg et al., 2014; Pérez‐Losada et al., 2009). The y‐nauplii are either planktotrophic (feeding) or lecithotrophic (nonfeeding), but the y‐cyprid is always nonfeeding. At least thirteen naupliar morphotypes are known to date, but only some of these have been correlated with y‐cyprids. This not only challenges adequate taxonomic classification but it also highlights a considerable knowledge gap on the lifecycle of y‐larvae and the structural variation in y‐cyprids.

The naupliar body consists of a cephalic anterior part, covered by the dorsal head shield, and a posterior part or hindbody. The y‐cyprid has a univalved carapace that only partially covers the larval body, six pairs of natatory thoracopods, a segmented thorax, and a limbless abdomen terminating with a telson with furcal rami (Figure 1). The dorsal side of the naupliar head shield, the “trunk,” the carapace, and the telson of the y‐cyprid have a surface pattern of reticulated cuticular ridges, which together form a series of interconnected plates or “facets”.

Rejecting an informal taxonomy for y‐larvae, Itô (1985) proposed the new genus *Hansenocaris* for his three new species (*H. pacifica*, *H. rostrata*, and *H. acutifrons*) described on the basis of their respective y‐cyprids. Five other new species of *Hansenocaris* were described later, *H. tentaculata* Itô, 1986, and *H. furcifera* Itô, 1989, from coastal waters of Japan, *H. itoi* Kolbasov & Høeg, 2003, from the White Sea (Figure 1a), *H. papillata* Kolbasov & Grygier, 2007 from coastal waters of Indonesia (Figure 1c) and *H. spiridonovi* Kolbasov, Savchenko, & Høeg, 2021 from Azores (Figure 1d). Additional six species of *Hansenocaris* were described on the basis of naupliar stages (Belmonte, 2005; Itô, 1985; Steuer, 1905; Swathi & Mohan, 2019), but they remain dubious, because they were not established on the basis of y‐cyprid morphology.

A recent study revealed seven naupliar instars in *Hansenocaris itoi* Kolbasov et Høeg, 2003, instead of the five that were previously supposed for the Facetotecta (Kolbasov et al., 2021b). This number of naupliar instars is unique not only for Facetotecta but also for Thecostraca and Hexanauplia as well.

Itô and Takenaka (1988), Itô (1989), Grygier (1987), Høeg and Kolbasov (2002), Kolbasov et al. (2007) and Kolbasov, Savchenko, and Høeg (2021) studied various aspects of the external and internal morphology of cyrpidoform larvae of Facetotecta in detail and discussed their relationships with other crustaceans.

Two informal morphological groups of facetotectan y‐cyprids were recognized by Kolbasov and Høeg (2003). The first, the “*Hansenocaris pacifica* group,” includes y‐cyprids with a long carapace with a round anterior end and sharp, laterally elongated posterior margins, and curved antennular hooks. This group includes all the Atlantic y‐cyprids, *H. itoi*, and also *H. pacifica* representing a type species of genus *Hansenocaris*, *H. furcifera*, and probably *H. papillata*. Y‐cyprids of the other group have a shorter head shield, often with an elongate and sharp anterior end, and supposedly lacking curved antennular hooks. *Hansenocaris rostrata*, *H. acutifrons*, and *H. tentaculata* belong to this group. This latter grouping has hardly any taxonomic value because of the very distinct morphology of *H. tentaculata* (e.g., the two‐segmented abdomen, instead of a four‐segmented one) compared to other Facetotecta.

Our studies of different y‐cyprids also revealed that some forms have labrum with numerous small spines instead of five long spines or lacking labrum at all (own unpublished data). These facts (different segmentation of abdomen, presence/absence of labrum, different morphology of antennules, labrum, paraocular process, thoracopods, and telson) indicate on the presence of several separate genera instead a single *Hansenocaris*. Thus, the known morphological variation of y‐cyprids challenges the concept of *Hansenocaris* and begs the question what the limits of the genus and the “bauplan” of y‐cyprids is?

**FIGURE 2:**
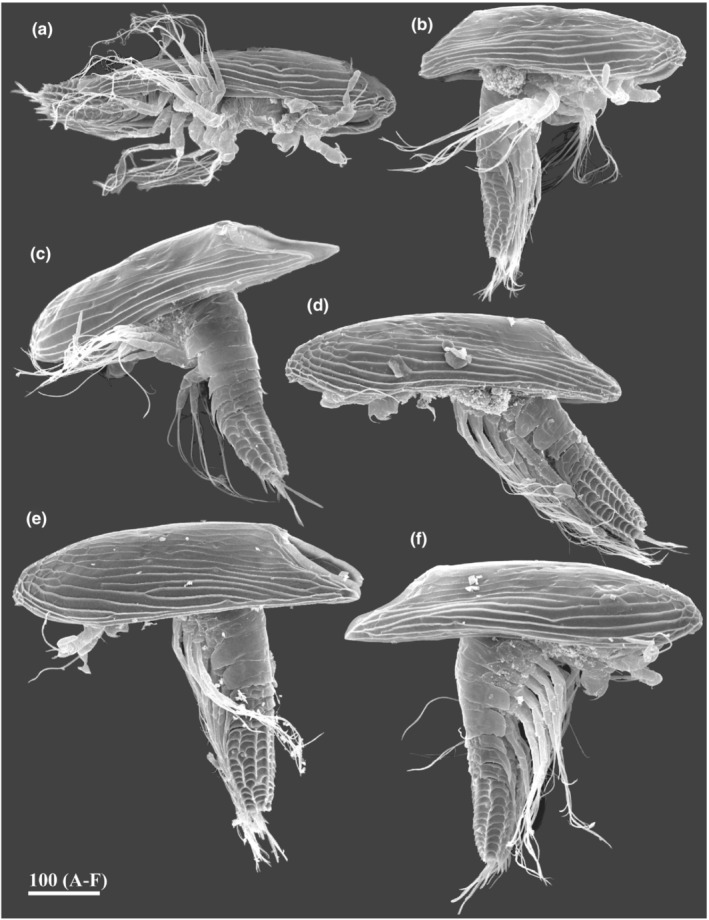
Y‐cypris larvae off north East Kamchatka, general view (a—Ventrolateral side, b–f Lateral side, SEM). (a–f) Y‐cypris larvae 1–6 respectively. Scale bar in μm.

Here, we describe in detail the morphology of six y‐cyprids from Kamchatka, Russia (Figure 2), and supplement with data on the morphology of other species and specimens studied by us (Figure 1). These y‐cyprids from Kamchatka belong to the “*Hansenocaris pacifica*”‐group, and may represent at least three different species. We systematically employ scanning electron microscopy to describe the morphology of all external structures of y‐cyprids. We reveal several morphological features that separate the “true” genus *Hansenocaris* from other representatives of Facetotecta and describe common characters for all y‐cyprids including six pairs of the lattice organs instead five pairs, which is presently considered as a ground pattern for all Thecostraca.

## 2 MATERIAL AND METHODS

The material was obtained in 2019 during the survey of the plankton collections of Zoological Institute RAS (St.‐Petersburg) and included six specimens of different y‐cyprids (Figure 2) collected off the Cape Africa, East Kamchatka (56°10′48″N, 163°22′12″E, 15–100 m). The study area is an open coastal part of the Bering Sea, approximately 2 km from the coast. All larvae were captured with a 72 μm mesh Juday plankton net No. 38, with 37 cm mouth opening. All material was preserved in 4% formalin and not suitable for molecular barcoding analysis, therefore we avoided describing new species. All cypridiform larvae were studied with an Olympus BX 43 light microscope and SEM. For SEM, the cypridiform larvae were postfixed in 2% OsO_4_ for 2 h, dehydrated in ethanol and acetone, and critically point dried by CO_2_, sputter‐coated with platinum–palladium and examined on JEOL JSM‐6380LA scanning electron microscope at operating voltages of 15–20 kV at the Laboratory of Electronic Microscopy of Moscow State University, Russia. Resulting photographs and photoplates were edited and assembled in CorelDraw X3 Graphics Suite.

We compare our SEM data obtained for cypridiform larvae to those from other locations: *H. itoi* from the White Sea (see Kolbasov & Høeg, 2003), *H. papillata* from coastal waters off Indonesia (see Kolbasov et al., 2007), *H. spiridonovi* from Azores (see Kolbasov et al., 2021a), and two undescribed species from Taiwan and abyssal Kuril‐Kamchatka Trench.

### 2.1 Morphological characters and taxonomical prospectus

In this study, we examined the following morphological characters (see also Table 1).

**TABLE 1:** The main characters of studied y‐cyprids and the described species of Facetotecta

Species and studied y‐cyprids	TL, μm	CL/TL	PL	Length of carapace	Anterior end of carapace	Sculpture of carapace	Paraocular process compared with a1	NF	ACL	Aesthetasc of a1	Labrum	Abdominal segments (excluding telson)	NTS	Furcal rami	Distribution and depth (m)
*H. acutifrons*	370	0.57	1/14	Reaching 4th thoracomere	Produced, sharp	Cuticular ridges reduced (very faint); no perforations	With equal protrusions, shorter than a1	9	Absent	Narrow, not bipartite	Reduced to blunt swelling	3 equal segments without long pleural extensions	6	Elongated, probably unsegmented, with 2 lanceolate setae	Southeast Japan, 0–5 m
*H. furcifera*	420	0.8	1/5	Reaching 6th thoracomere	Rounded	Cuticular ridges clear in anterior part, reduced in middorsal; no perforations	With equal protrusions, shorter than a1	9	Present, comparable with 2nd segment of a1	Narrow, not bipartite	Developed, with 5 spines	3 equal segments; 2nd, 3rd with long pleural extensions	5	Small, ‘two‐annulated', probably unsegmented, with 3 lanceolate setae	Southeast Japan, probably Sea of Japan, 0–5 m
*H. itoi*	570	0.8	1/4	Reaching telson	Rounded	Cuticular ridges conspicuous in anterior part, reduced in midposterior; no perforations	With equal protrusions, shorter than a1	14–15	Present, comparable with 2nd segment of a1	Swollen, bipartite	Developed, with 5 spines	3 equal segments with long pleural extensions	5	Small, unsegmented, with 3 lanceolate setae and tiny seta, with basal papilla	White Sea, probably Barents Sea and Kara Sea, 0–40 m
*H. pacifica*	460	0.6	1/12	Reaching 5th thoracomere	Rounded	Cuticular ridges clear and delicate; no perforations	With equal protrusions, shorter than a1	10–14	Present, comparable with 2nd segment of a1	Narrow, not bipartite	Developed, with 5 spines	3 equal segments without long pleural extensions	5	Small, unsegmented, with 3 lanceolate setae	Southeast Japan, 0–5 m
*H. papillata*	430	0.56	1/9	Reaching 4th thoracomere	Rounded	Cuticular ridges delicate; no perforations	N/o	N/o	Present, comparable with 2nd segment of a1	Narrow, not bipartite	Developed, with 5 spines	3 segments; 3rd segment significantly shorter than 1st and 2nd; without pleural extensions	5	Small, unsegmented, with 3 lanceolate setae	Eastern Indonesia. 2.2 m
*H. rostrata*	380	0.54	1/10	Reaching 4th thoracomere	Produced	Cuticular ridges reduced (very faint); no perforations	With equal protrusions, shorter than a1	N/o	Absent	Narrow, not bipartite	Developed, with 5 spines	3 equal segments without long pleural extensions	5	Elongated, unsegmented, with 3 lanceolate setae	Southeast Japan, 0–5 m
*H. spiridonovi*	509	0.64	1/8	Reaching 6th thoracomere	Rounded	Cuticular ridges developed, conspicuous; no perforations	N/o	>2	Present, comparable with 2nd segment of a1	Narrow, not bipartite	Developed, with 5 spines	3 equal segments; 2nd, 3rd with long pleural extensions	4	Small, two‐joined, probably unsegmented, with 3 lanceolate setae, with basal papilla	Azores, 0–2 m
*H. tentaculata*	350	0.5	1/8	Reaching 3rd thoracomere	Slightly produced, sharp	Cuticular ridges reduced (very faint); no perforations	With very long, unequal, protrusions, longer than a1	N/o	Absent	Narrow, not bipartite	Developed, with 5 spines	1 segment without long pleural extensions	0	Small, unsegmented, with 3 lanceolate setae	Southeast Japan, 0–5 m
Kamchatka y‐cypris 1	560	0.8	1/4	Reaching telson	Rounded	Cuticular ridges clear; no perforations	With equal protrusions, shorter than a1	N/o	Present, comparable with 2nd segment of a1	Swollen (bulbous), bipartite	Developed, with 5 spines	3 equal segments with long pleural extensions	5	Small, unsegmented, with 3 lanceolate setae and tiny seta, with basal papilla	East Kamchatka, 15–100 m
Kamchatka y‐cypris 2	590	0.64	1/5	Reaching 2nd abdominal segment	Rounded	Cuticular ridges clear, feeble on dorsal side; no perforations	With equal protrusions, shorter than a1	13	Present, comparable with 2nd segment of a1	Swollen (bulbous), bipartite	Developed, with 5 spines	3 equal segments; 2nd, 3rd with long pleural extensions	5	Small, unsegmented, with 3 lanceolate setae and tiny seta, with basal papilla	East Kamchatka, 15–100 m
Kamchatka y‐cypris 3	585	0.75	1/3	Reaching telson	Rounded	Cuticular ridges clear, reduced on dorsal side; no perforations	N/o	N/o	Present, comparable with 2nd segment of a1	Narrow, not bipartite	Developed, with 5 spines	3 equal segments with long pleural extensions	5	Small, unsegmented, with 3 lanceolate setae and tiny seta, with basal papilla	East Kamchatka, 15–100 m
Kamchatka y‐cypris 4	648	0.71	1/5	Reaching telson	Rounded	Cuticular ridges clear, reduced in midposterior; no perforations	With equal protrusions, shorter than a1	10	Present, comparable with 2nd segment of a1	Swollen (bulbous), bipartite	Developed, with 5 spines	3 equal segments with long pleural extensions	5	Small, unsegmented, with 3 lanceolate setae and tiny seta, with basal papilla	East Kamchatka, 15–100 m
Kamchatka y‐cypris 5	605	0.76	1/4	Reaching telson	Rounded	Cuticular ridges clear, feeble in midposterior; no perforations	With equal protrusions, shorter than a1	11	Present, comparable with 2nd segment of a1	Swollen (bulbous), bipartite	Developed, with 5 spines	3 equal segments with long pleural extensions	5	Small, unsegmented, with 3 lanceolate setae and tiny seta, with basal papilla	East Kamchatka, 15–100 m
Kamchatka y‐cypris 6	640	0.73	1/4	Reaching telson	Rounded	Cuticular ridges clear, reduced in middorsal; no perforations	With equal protrusions, shorter than a1	10	Present, comparable with 2nd segment of a1	Narrow, not bipartite	Developed, with 5 spines	3 equal segments; 2nd, 3rd with long pleural extensions	5	Small, unsegmented, with 3 lanceolate setae and tiny seta, with basal papilla	East Kamchatka, 15–100 m
Y‐cypris from Kuril Trench	623	0.83	1/5	Reaching telson	Rounded	Cuticular ridges conspicuous, developed; with dense, numerous perforations	N/o	N/o	Present, larger than 2nd segment of a1	Narrow, not bipartite	Absent	3 equal segments; 2nd, 3rd with long pleural extensions	4	Small, unsegmented, with 3 lanceolate setae, with basal papilla	Kuril‐Kamchatka Trench, 3000–5900 m

Abbreviations: a1—antennule; ACL—antennular claw; CL/TL—ratio of length of carapace to total length; NF—number of filaments in the postocular filamentary tuft; N/o—not observed; NTS—number of serrate spines along the posterioventral margin of the telson; PL—ratio of length of posteriolateral corners of carapace to length of carapace; TL—total length.

*Total length of y‐cypris* was measured from anterior most end of the carapace to the posterior end of furcal rami excluding setae.

**FIGURE 3:**
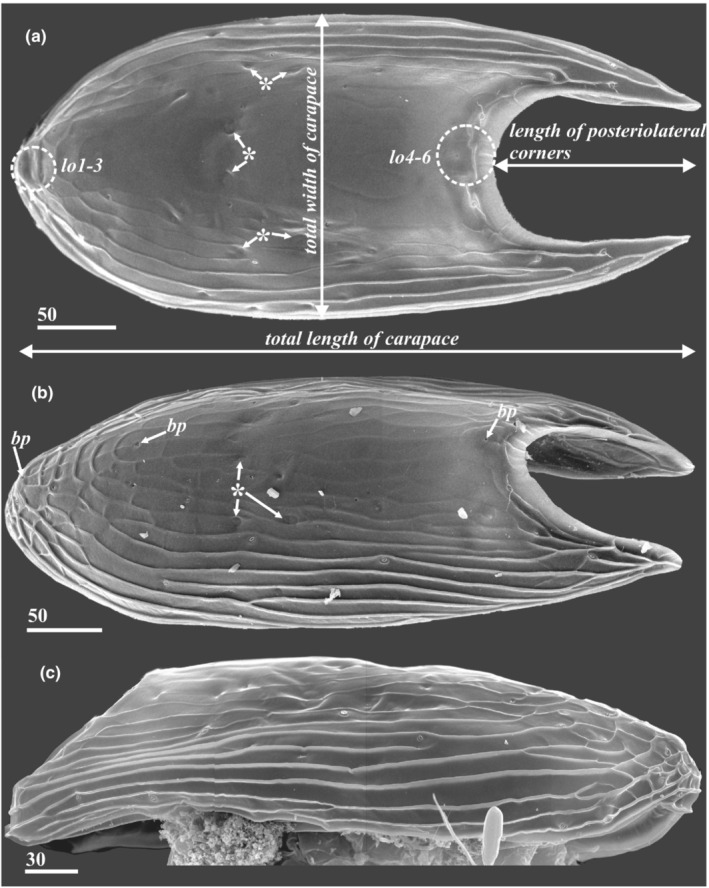
Morphology of carapace of y‐cypris larvae showing different degree of cuticular ridges development and proportions/length of posteriolateral corners (a, b—Dorsal side, c—Lateral side. a—y‐cypris larva 3; b—y‐cypris larva 5; c—y‐cypris larva 2. SEM). Locations of lattice organs indicated by round dotted outlines; scars/pits of muscle insertions indicated by asterisks. Major carapace dimensions indicated in ‘A'. Abbreviations: *bp*—big central unpaired pores; *lo1‐6*—lattice organs. Scale bars in μm.

**FIGURE 4:**
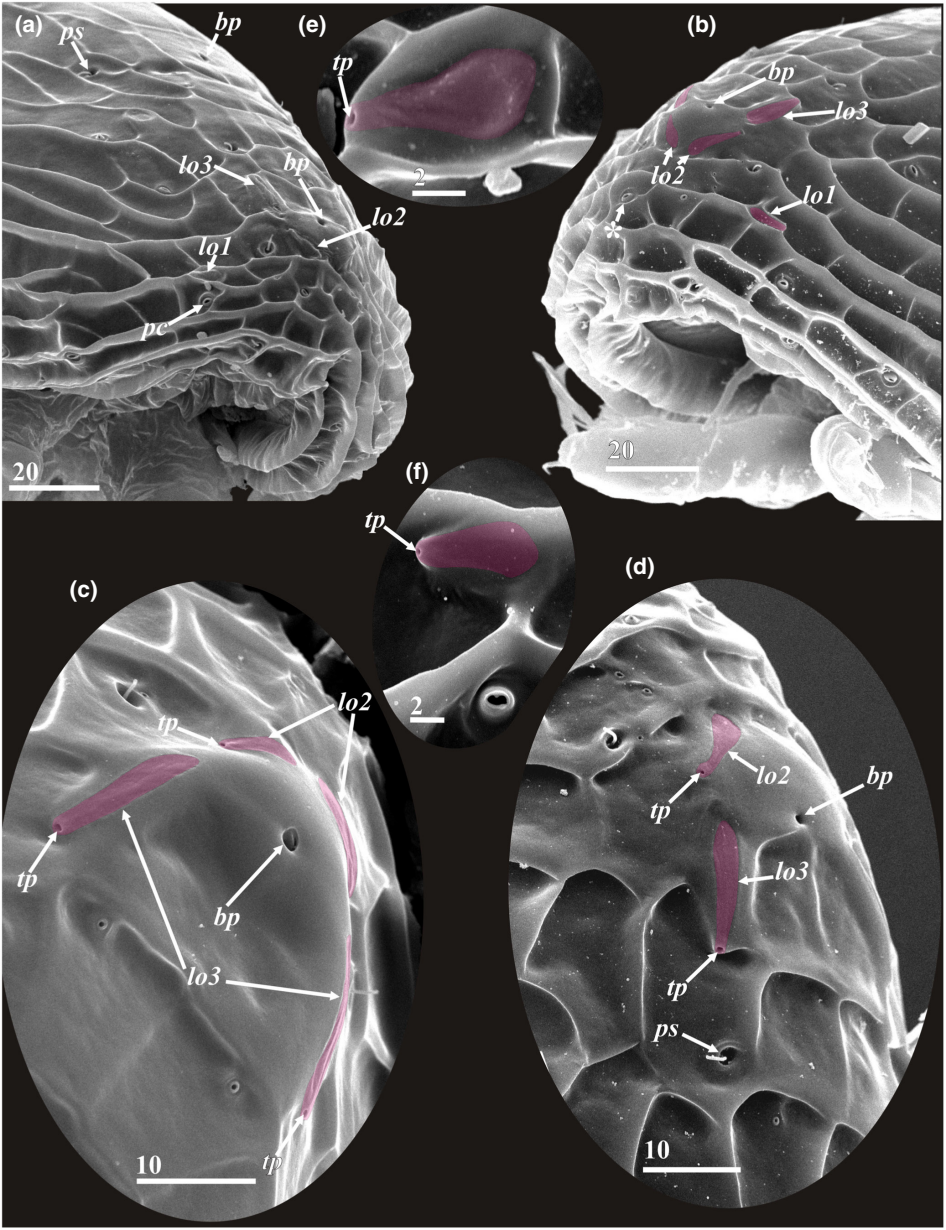
Anterior lattice organs of y‐cypris larvae of Facetotecta (a, e—y‐cypris larva 2; b, d—y‐cypris larva 4; c—y‐cypris larva 3; f—y‐cypris larva 6. SEM). Lattice organs indicated by magenta color in b–e. (a, b) Locations of anterior lattice organs (*lo1‐3*) on carapace, anteriormost unpaired pore with cuticular rim indicated by asterisk. (c, d) Locations of lattice organs 2 and 3. (e, f) Lattice organ 1. *bp*—big central unpaired pores; *lo1‐3*—lattice organs; *pc*—pore with cuticular rim; *ps*—pit/pore with seta inside; *tp*—terminal pore of lattice organ. Scale bars in μm.

**FIGURE 5:**
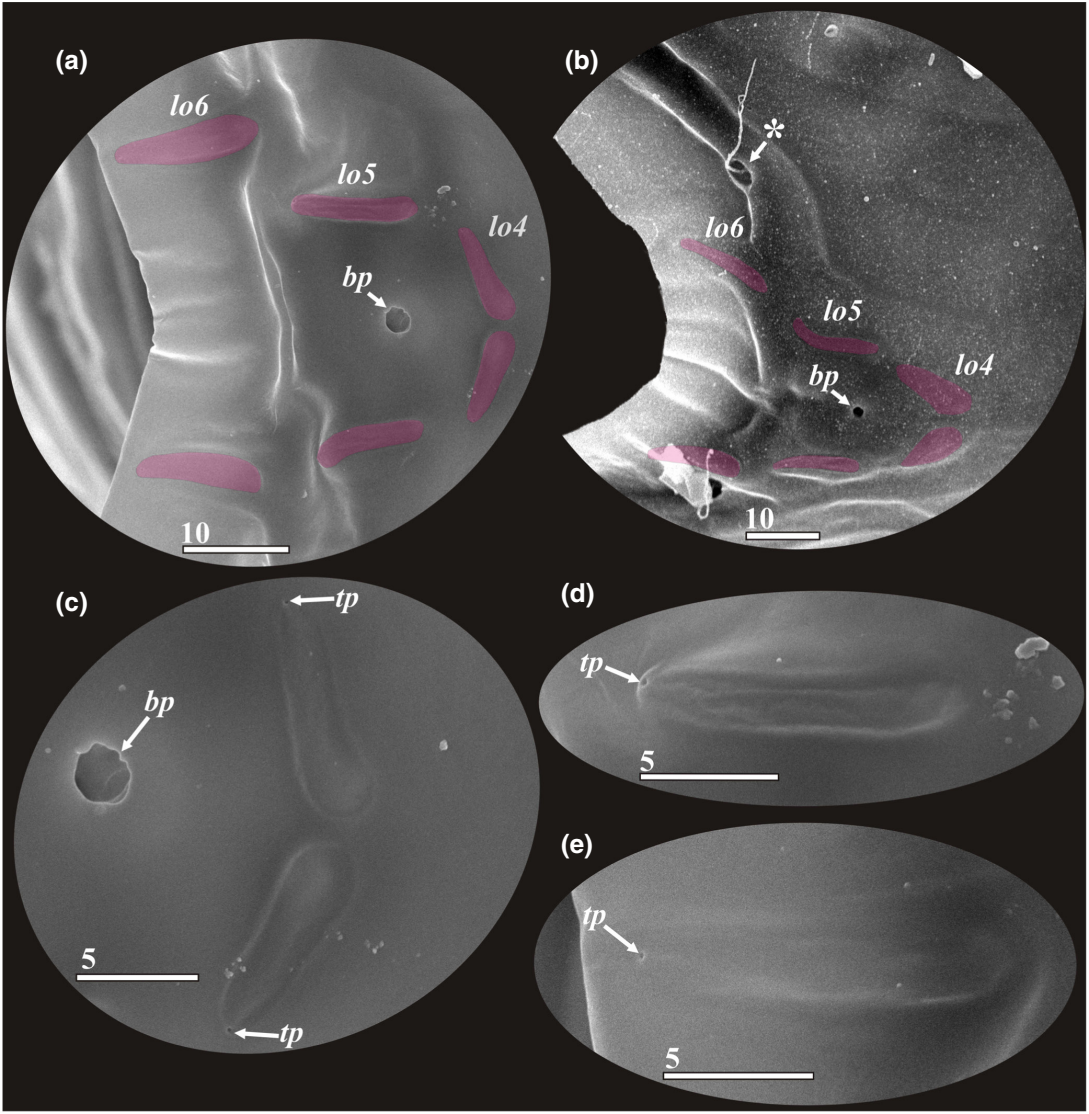
Posterior lattice organs of y‐cypris larvae of Facetotecta (a, c–e—y‐cypris larva 3; b—y‐cypris larva 5. SEM). (a, b) Locations of posterior lattice organs (*lo4‐6*) on carapace; lattice organs indicated by magenta color, large pit/pore with long seta inside indicated by asterisk. (c) Lattice organs 4. (d, e) Lattice organs 5 and 6 respectively. *bp*—big central unpaired pores; *lo4‐6*—lattice organs; *tp*—terminal pore of lattice organ. Scale bars in μm.

*Carapace* (Figure 3): Total length was measured from anteriormost to posteriormost ends of carapace (Figure 3a). Total width represents the largest width of carapace (Figure 3a). Length of posteriolateral corners—size of posterior notch of carapace (see Figure 3a). Development of posteriolateral corners of carapace—i.e., to which trunk segment they reach. Form of posteriolateral corners of carapace: sharp (Figures 2c and 3a,b); blunt or rounded (Figures 2b,d and 3c). Form of anterior end of carapace: rounded or slightly pointed (Figure 3); sharp and elongated (strongly produced see figures in descriptions of *H. acutifrons*, *H. rostrata*, and *H.tentaculata* in Itô (1985, 1986)). Development of cuticular ridges of carapace: feeble or reduced on the whole surface (Figure 1c); feeble or reduced on dorsal side (Figure 3a); developed (Figure 1b,d). Development of cuticular ridges in anterior part of carapace: clear (Figures 1a,b,d and 3a,b,c); unclear or reduced (Figure 1c). Surface of carapace: without “perforations” (Figures 1a,c,d, 2 and 3); perforated with numerous small pits (Figures 1b and 11d). Number of unpaired central pores (with/without cuticular rim; Figures 3b, 4 and 5, “*bp*”). Number of paired pores with cuticular rim (Figures 4 and 11, “*pc*”). Number of paired rounded pores with seta inside (Figures 4, 5 and 11, “*ps*”). Number and form of the lattice organs (Figures 4, 5 and 11).

**FIGURE 6:**
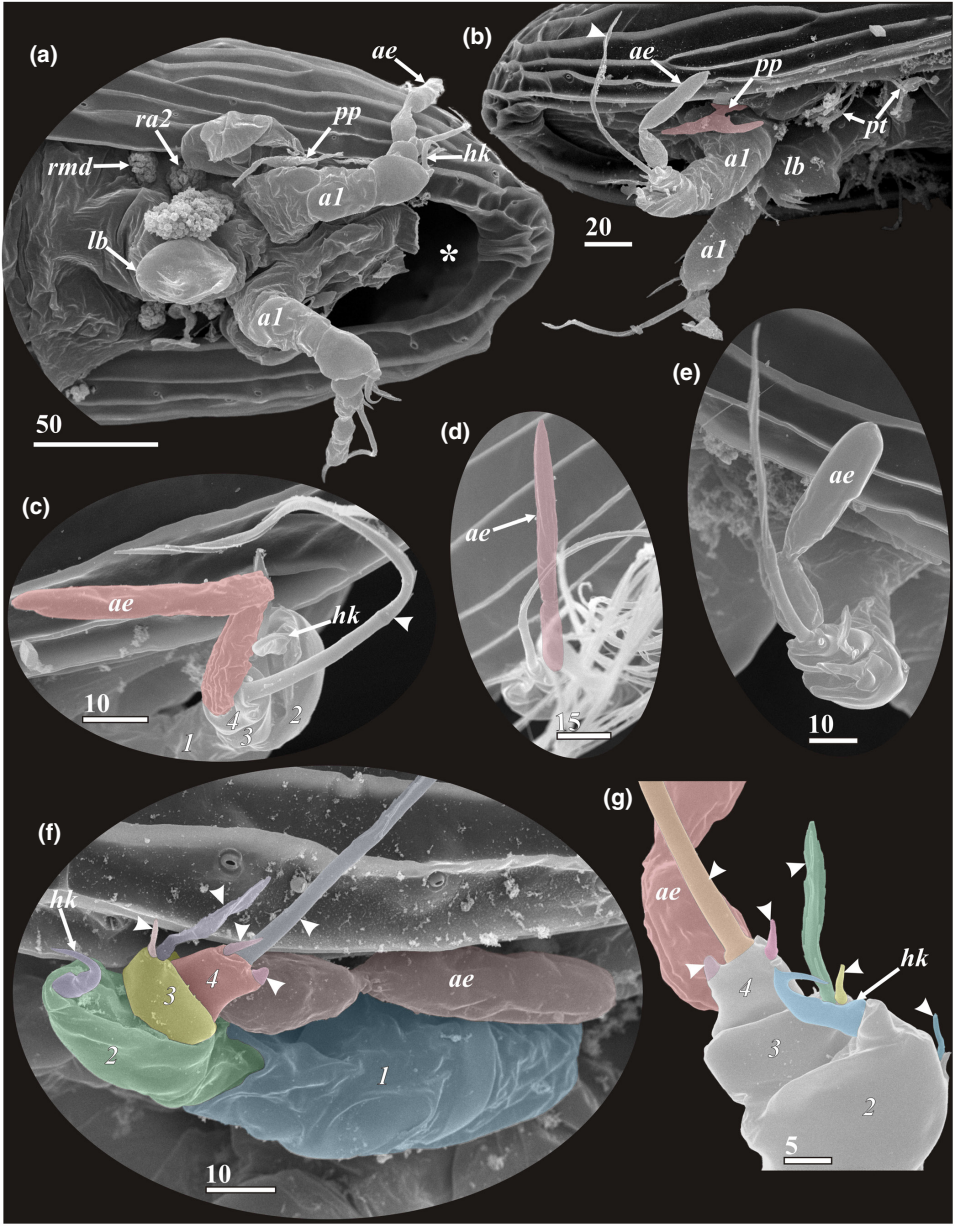
Cephalic appendages and structures of y‐cypris larvae of Facetotecta (a—y‐cypris larva 1; b—y‐cypris larva 5; c—y‐cypris larva 6; d—y‐cypris larva 3; e, g—y‐cypris larva 2; f—y‐cypris larva 4. SEM). (a) Anterior part of y‐cypris exuvium, ventral side (ypsigon exit opening in anterior end indicated by asterisk). (b) Anterior part, lateroventral view (paraocular process colored in red; thin and long seta of 4th antennular segment indicated by arrowhead). (c–e) distal parts of antennules (narrow, ribbon‐shaped aesthetascs without constriction couloured in red in ‘C' and ‘D'; segments numbered in Arabic in ‘C'). (f) Antennule (segments, setae and hook indicated by different colors; segments numbered in Arabic, setae indicated by arrowheads). (g) Distal half of antennule (setae and hook indicated by different colors; segments numbered in Arabic, setae indicated by arrowheads). *a1*—antennules; *ae*—aesthetasc; *hk*—hook/claw of 2nd antennular segment; *lb*—labrum; *pp*—paraocular process; *pt*—postocular filamentary tuft; *ra2*—rudiment of antennae; *rmd*—rudiment of mandible. Scale bars in μm.

**FIGURE 7:**
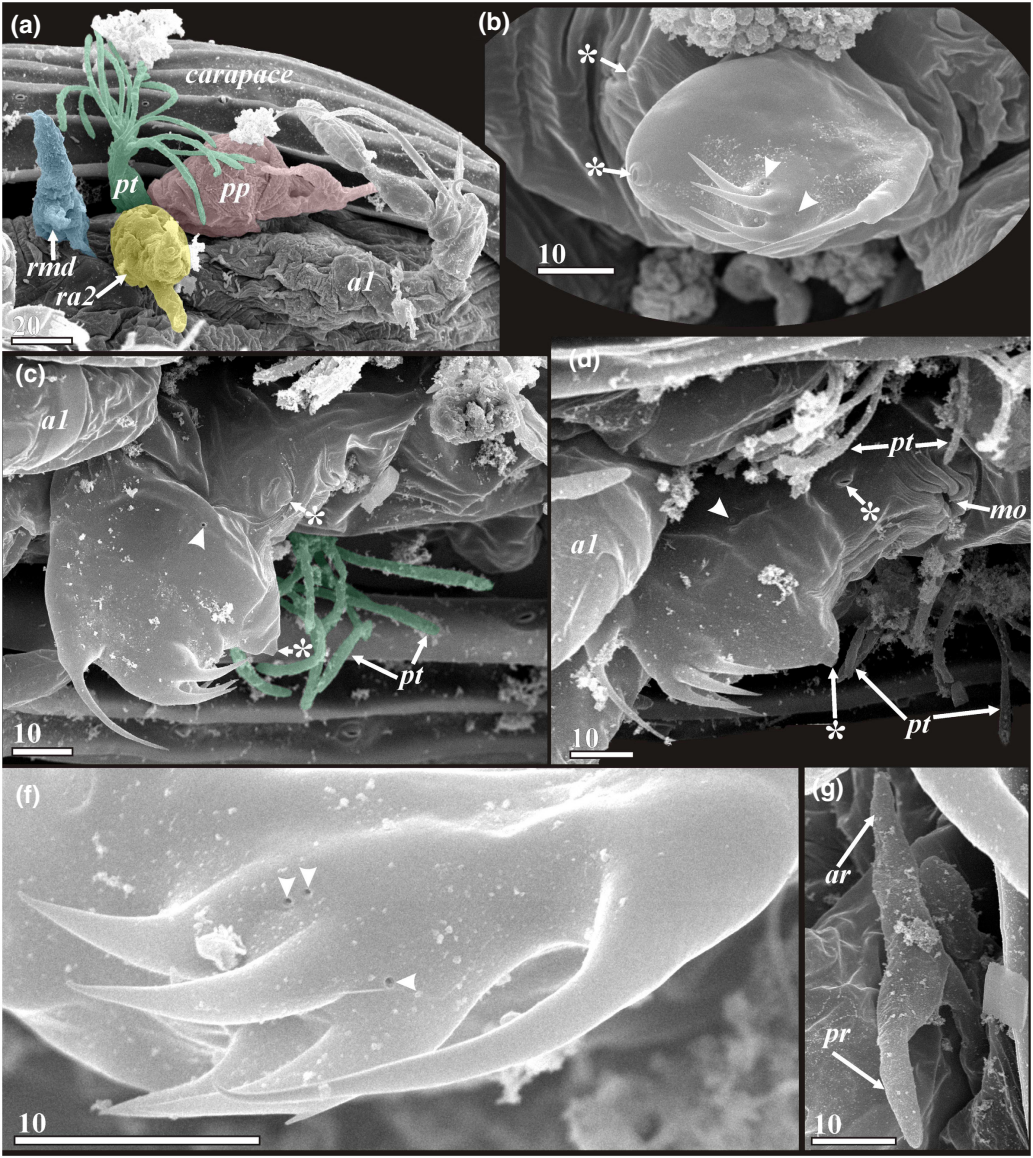
Cephalic appendages and structures of y‐cypris larvae of Facetotecta (a—*H. itoi*; b—y‐cypris larva 1; c—y‐cypris larva 4; d, g—y‐cypris larva 5; f—y‐cypris larva 2. SEM). (a) Anterior part with antennule, paraocular process (colored in red; basal bulbous part may represent lower half of compound eye), postocular filamentary tuft (colored in green) and rudiments of antenna (colored in yellow) and mandible (colored in cyan). (b) Labrum, ventral view (big pores indicated by asterisks, tiny pores indicated by arrowheads). (c) Labrum (big pores indicated by asterisks, tiny pores indicated by arrowheads) and postocular filamentary tuft (filaments colored in green), lateral view. (d) Labrum and postocular filamentary tufts (big pores indicated by asterisks, tiny pores indicated by arrowheads). (f) Spines in distal part of labrum (tiny pores indicated by arrowheads). (g) Paraocular process. *a1*—antennules; *ar*—anterior ramus of paraocular process; *mo*—mouth opening; *pp*—paraocular process; *pr*—posterior ramus of paraocular process; *pt*—postocular filamentary tuft; *ra2*—rudiment of antennae; *rmd*—rudiment of mandible. Scale bars in μm.

*Cephalic appendages* (Figures 6 and 7). Size, number, and position of antennular setae (Figure 6c–g). Armament of antennular segment 2: with hook (Figure 6); without hook. Relative size of antennular hook: comparable with segment 2 (Figure 6); significantly larger than segment 2. Form of antennular aesthetasc: bulbous, not bipartite; bulbous and bipartite—constricted at mid‐length (Figure 6a,b,e–g); narrow, not bipartite (Figure 6c,d). Size, number and position of antennular setae (Figure 6f,g). Labrum: developed (Figures 6a,b and 7b,d); reduced. Number of spines of labrum: 3; 4; 5 (Figure 7f); more than 5. Relative size and form of paraocular process: with equal distal protrusions and shorter than antennule (Figures 6b and 7g); with very long, unequal distal protrusions comparable or longer than antennule. Number of filaments in postocular filamentary tuft (Figure 7a,c).

**FIGURE 8:**
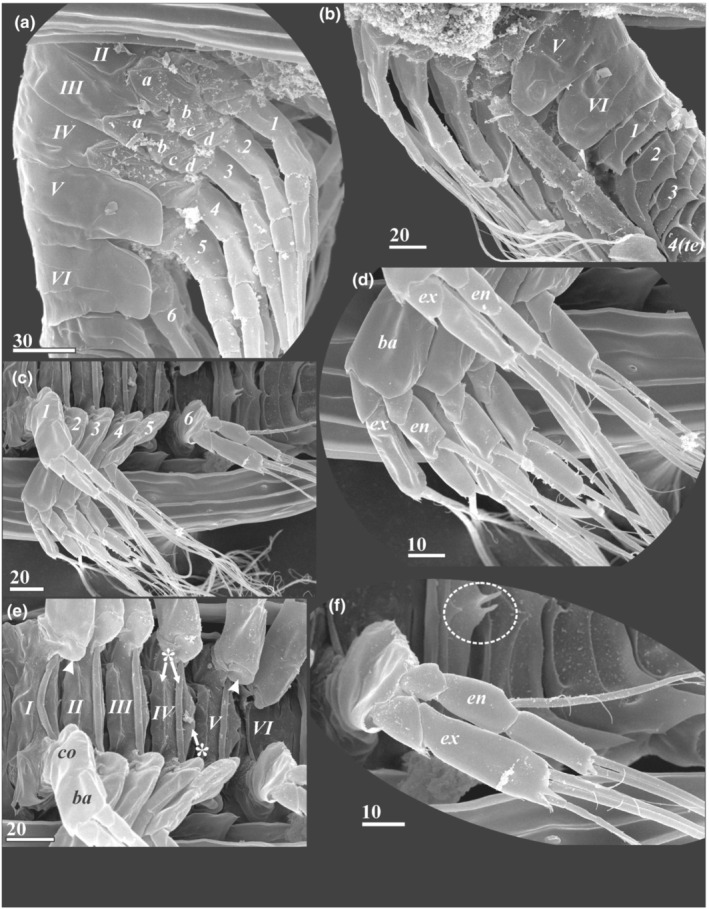
Trunk and thoracopods of y‐cypris larvae of Facetotecta (a—y‐cypris larva 6; b—y‐cypris larva 4; c–f—y‐cypris larva 1. SEM). (a) Free thoracic segments (numbered in Roman) and thoracopods (numbered in Arabic; ‘*a, b, c, d*'—proximal ‘coxicules’), lateral view. (b) Last thoracic (numbered in Roman) and abdominal segments (numbered in Arabic), lateral view. (c) Right thoracopods (numbered in Arabic), ventral view. (d) Left thoracopods 1–5, ventral view. (e) Sternites of thoracomeres (numbered in Roman; sclerotized elements of sternites indicated by asterisks) and bases of thoracopods (basal coxal sclerites indicated by arrowheads). (f) Thoracopod 6 (putative vestige of penis indicated by oval dotted outline). *ba*—basis; *co*—coxa; *en*—endopod; *ex*—exopod; *te*—telson. Scale bars in μm.

*Thorax* (Figures 1, 2 and 8a,b): Form of pleural extensions in thoracomeres 5 and 6: rounded (Figures 1a, 2d and 8b); rectangular/trapezoid with sharpen/rounded posteriolateral corners (Figures 1c,d, 2c,e,f and 8a).

*Segmentation of thoracopods* (Figure 8): T1 exopod and endopod segmentation (2 + 2 or 2 + 1). T2‐5 exopod and endopod segmentation (2 + 3 or 2 + 2, or T2 has 2 + 1 segmentation). T6 exopod and endopod segmentation (2 + 3 or 2 + 2). See further explanations in “Results” (“Thorax and thoracopods”).

**FIGURE 9:**
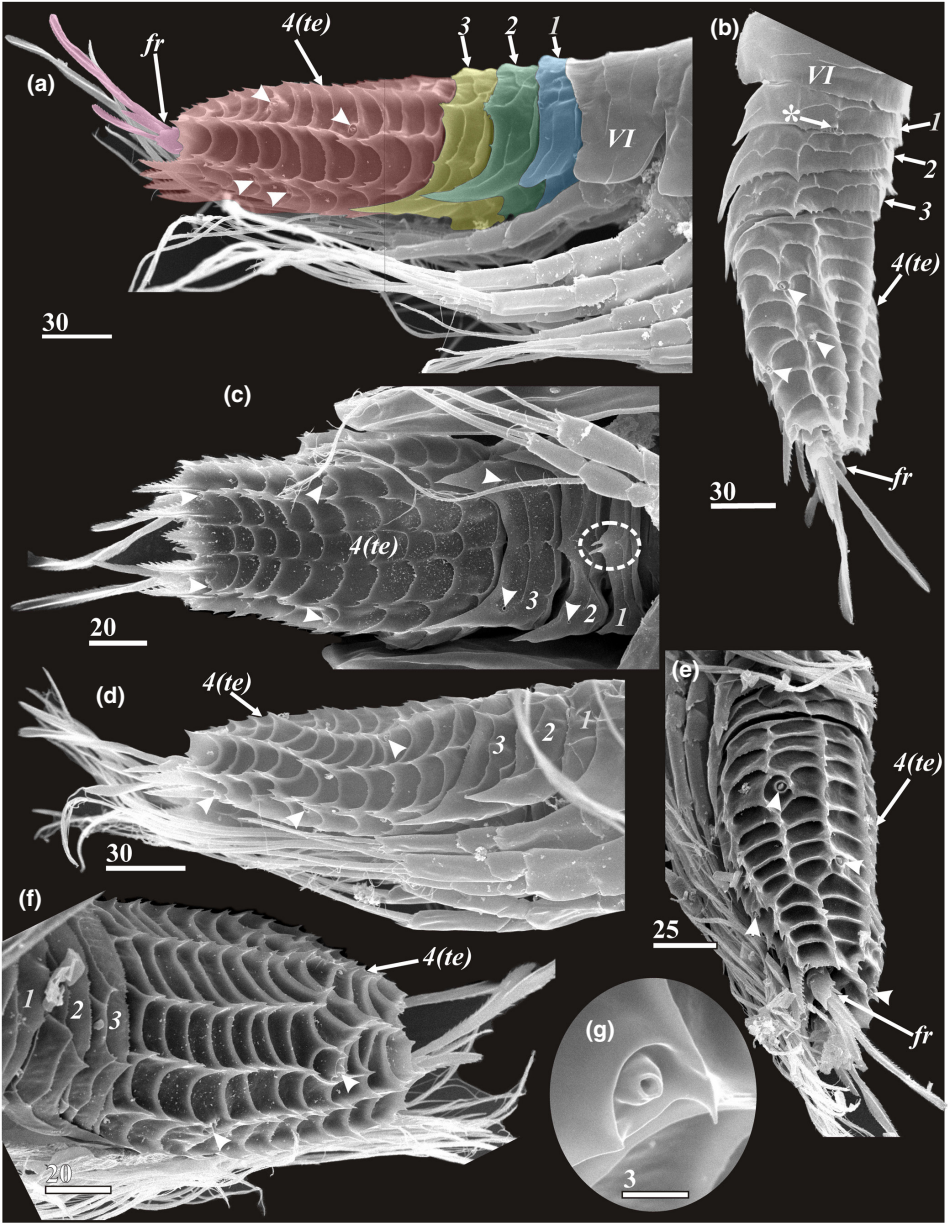
Abdomen of y‐cypris larvae of Facetotecta (a—y‐cypris larva 6; b—y‐cypris larva 3; c—y‐cypris larva 1; d, g—y‐cypris larva 2; e—y‐cypris larva 5; f—y‐cypris larva 4. SEM). (a) Abdomen, lateral view (abdominal segments and furcal ramus indicated by different colors; thoracic segment numbered in Roman, abdominal segments numbered in Arabic; telsonic pores indicated by arrowheads). (b, d, e) Abdomen, lateral view (thoracic segment numbered in Roman, abdominal segments numbered in Arabic; telsonic pores indicated by arrowheads; pit/pore with seta indicated by asterisk). (c) Abdomen, ventral view (abdominal segments numbered in Arabic; abdominal pores indicated by arrowheads; putative vestige of penis indicated by oval dotted outline). (f) Abdomen, dorsal view (abdominal segments numbered in Arabic; telsonic pores indicated by arrowheads). (g) Lateral telsonic pore. *fr*—furcal rami; *te*—telson. Scale bars in μm.

**FIGURE 10:**
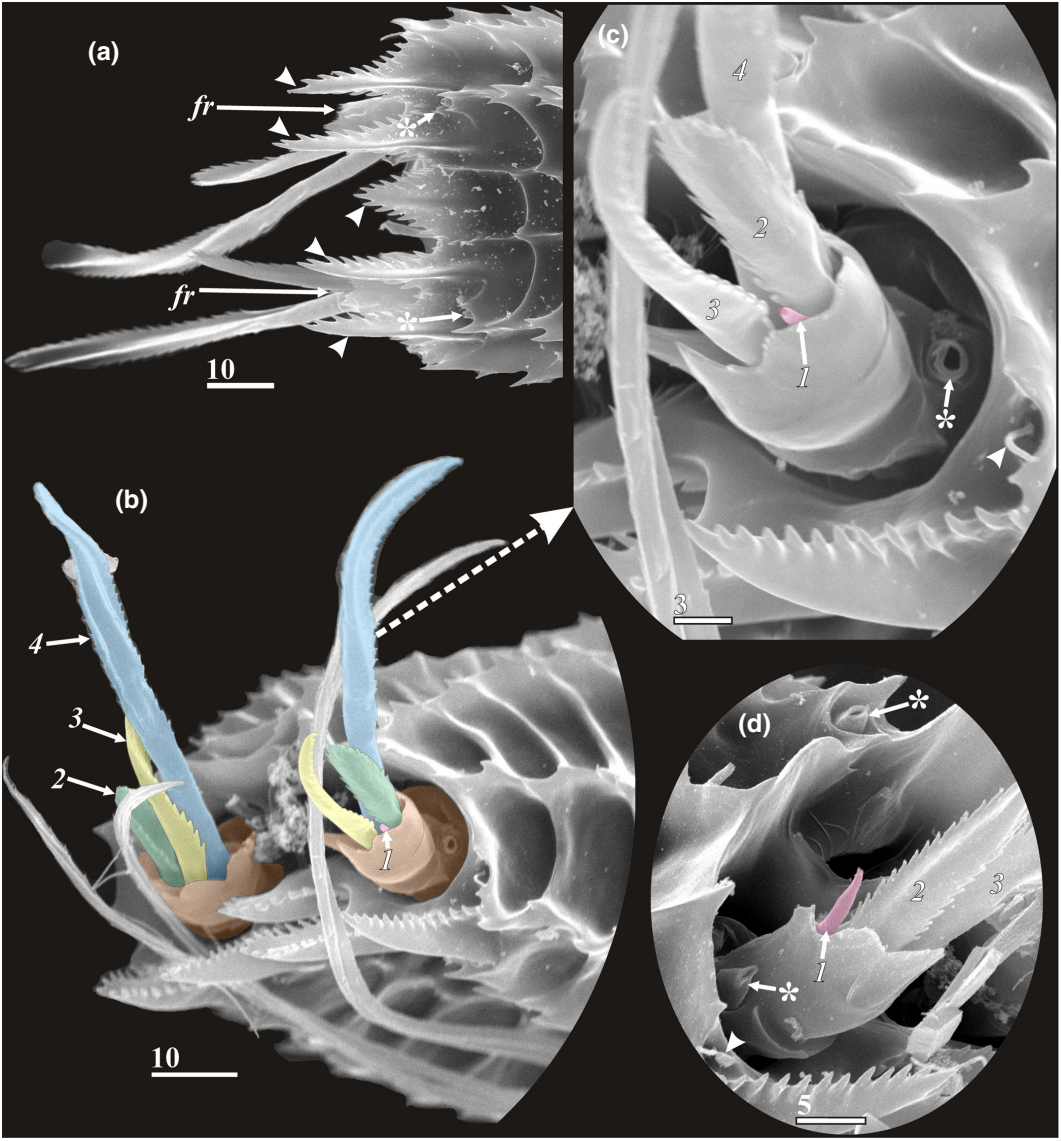
Furcal rami and abdomen of y‐cypris larvae of Facetotecta (a—y‐cypris larva 1; b, c—y‐cypris larva 6; d—y‐cypris larva 5. SEM). (a) Posterior margin of telson, ventral view (five ventral terminal telsonic spines indicated by arrowheads; telsonic pores indicated by asterisks). (b) Furcal rami (orange) with setae (numbered in Arabic and indicated by different colors). (c, d) Enlarged furcal ramus (setae numbered in Arabic, tiny seta colored in magenta; pores indicated by asterisks; pit with seta indicated by arrowhead). *fr*—furcal rami. Scale bars in μm.

*Abdomen and furcal rami* (Figures 9 and 10): Number of abdominal segments including telson: four segments (Figure 9); two segments (*H. tentaculata*). Size of first three abdominal segments: all segments of comparable size (Figure 9); third segment significantly smaller than others (Figure 1c). Size and form of pleural extensions of abdominal segments: all abdominal segments with long, spiniform posteriolateral pleural extensions (Figures 1a and 9b); first abdominal segment with short posteriolateral pleural extensions (Figure 1d); all abdominal segments with short posteriolateral pleural extensions (Figure 1c). Relative size of telson including posterioventral spines (ratio to the total length). Number of cuticular plates in dorsal, lateral, and ventral rows of telson (Figure 9). Number of serrate spines along posterioventral margin of telson (Figure 10a). Number and position of pores of telson (Figures 9 and 10a,c,d). Shape, size, and armament of furcal rami (Figure 10).

In having elongated carapace with rounded anterior end, four‐segmented abdomen with five serrated spiniform processes on posterioventral margin of telson, antennules with hook, and labrum with five spines, all studied y‐cyprids in the new material (Figure 2) belong to the “*Hansenocaris pacifica*”‐group. Y‐cyprids 1, 4, and 5 (Figure 2a,d,e) may be conspecific or closely related to *H. itoi* (Figure 1a) in having bipartite antennular aesthetasc, similar pattern and development of cuticular ridges of carapace, by form and size of posteriolateral corners of the carapace and structure of abdominal segments. Y‐cyprids 2, 3, and 6 (Figure 2b,c,f) may represent new species of Facetotecta but were preserved in 4% formalin and not suitable for molecular barcoding analyses. Therefore, we avoid describing them formally here. Although y‐cypris 2 also has bipartite aesthetascs, it differs from *H. itoi* and related specimens by sporting a relatively shorter carapace with short and blunt posteriolateral ends (Figures 2b and 3c). Y‐cyprids 3 and 6 are characterized by narrow aesthetascs without constriction (Figure 6c,d) and thus resemble *H. pacifica* and *H. furcifera* in this character. These y‐cyprids differ from *H. pacifica* and *H. furcifera* in having longer posteriolateral corners of carapace reaching abdomen and by the form of posteriolateral pleural extensions of the abdominal segments and the number of cuticular plates of the telson.

*H. pacifica* is a type species of the genus *Hansenocaris*, and the morphological diagnosis of this taxon should be based on the characters of y‐cyprids belonging to the “*Hansenocaris pacifica*” group including the Kamchatka y‐cyprids studied here.

## 3 RESULTS

### 3.1 Carapace and its structures (Figures 1, 2, 3, 4, 5 and 11, Table 1)

**FIGURE 11:**
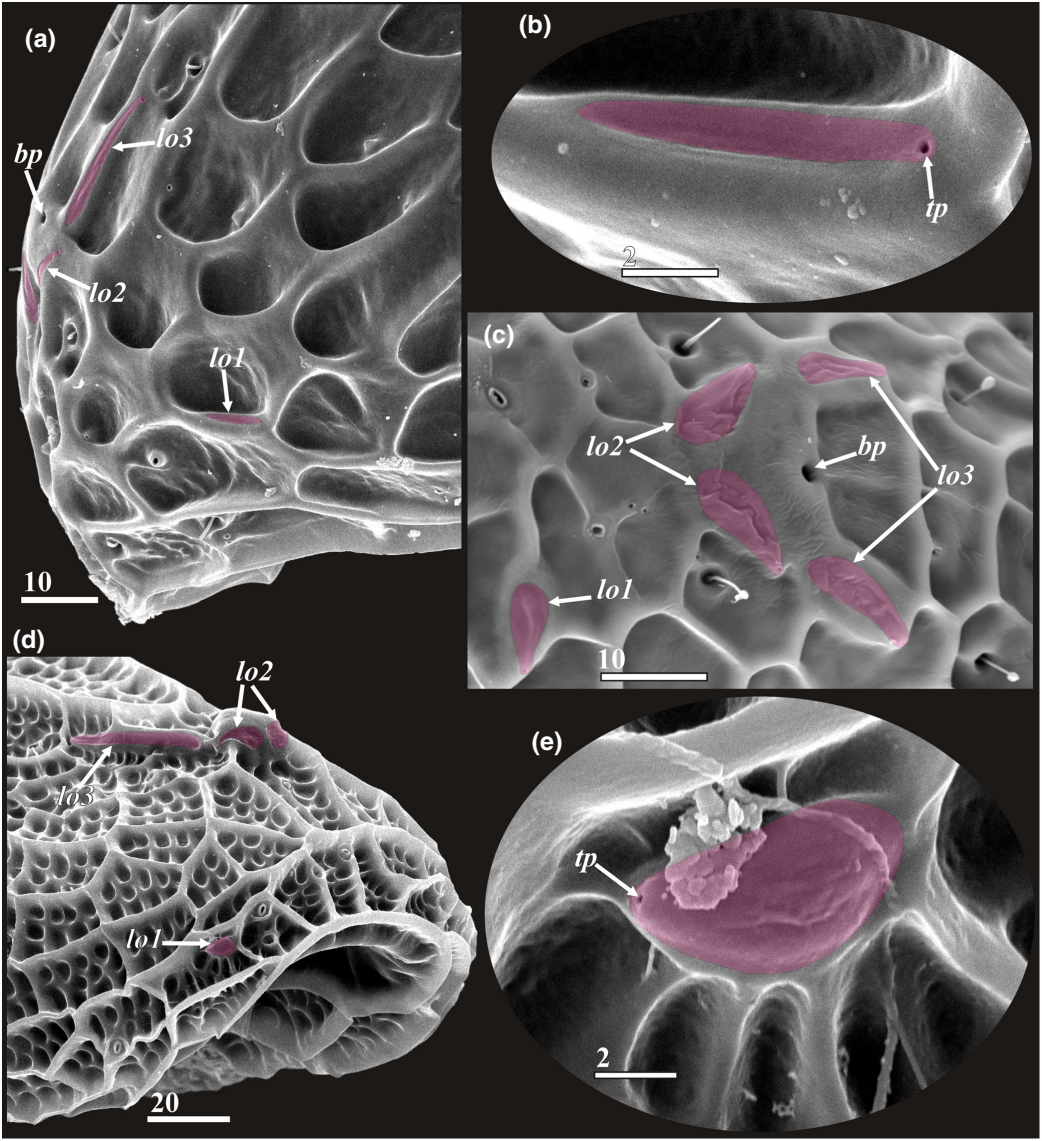
Anterior lattice organs of different y‐cypris larvae of Facetotecta (a, b—*H. spiridonovi* from subtropical Azores Islands; c—*H. itoi* from arctic White Sea; d, e—Y‐cypris larva from boreal Kuril‐Kamchatka trench. SEM. Lattice organs indicated by magenta color). (a, c, d) Locations of anterior lattice organs (a, d—Lateral view; c‐ dorsal view). (b, e) Lattice organ 1. *bp*—big central unpaired pores; *lo1‐3*—lattice organs; *tp*—terminal pore of lattice organ. Scale bars in μm.

The carapace of all y‐cyprids is univalved and resembles an inverted boat hull and only partially covers the larval body (Figures 1, 2 and 3). In most of the y‐cyprids studied, the cuticle of the carapace is not densely perforated with small pits (Figures 1a,c,d and 2). But such perforation is present in those from the Kuril‐Kamchatka Deep Trench (Figures 1b and 11d) and also in y‐cyprids from Bahamian waters (specimen no. 2 of Schram (1970), see figure 4a in Høeg & Kolbasov, 2002). The total length of studied y‐cyprids from Kamchatka varies from 560 to 648 μm and from 440 to 470 μm for their carapaces, with a carapace to length ratio at 0.64 to 0.8 (see Table 1 for other Facetotecta). The anterior end of the carapace in most studied y‐cyprids, including all from the “*Hansenocaris pacifica*”‐group, is rounded (Table 1; Figure 3a,b), while *H. acutifrons*, *H. rostrata*, and *H. tentaculata* possess produced and sharpened anterior ends (Table 1). The carapace of all y‐cyprids has posteriolateral outgrowths or corners of different length and form (Table 1; Figures 1, 2 and 3). These corners form a posterior notch of the carapace. The posteriolateral corners may be elongated (1/3–1/4 of length of carapace; Table 1; Figures 1a,b, 2c–f and 3a,b), moderate (1/5–1/8 of length of carapace; Table 1; Figures 1d, 2b and 3c), or short (1/9–1/14 of length of carapace; Table 1; Figure 1c). Their edges may be pointed (Figures 1b and 2c) or rounded/blunt (Figures 1a,c,d, 2b,d–f and 3c).

In the Kamchatka specimens long longitudinal cuticular ridges ornament the lateral sides (Figure 2). Cuticular ridges are more developed in the anterior end where they form several plates (Figures 2 and 3), whereas the medio‐posterior or whole medio‐dorsal area of the carapace has feeble delicate or lacks any ridges (Figure 3a,b). In other y‐cyprids the cuticular ridges of the carapace may have the same pattern and development (Table 1; Figure 1a), being delicate or reduced (Table 1; Figure 1c), or strongly developed on the whole surface of carapace (Table 1; Figure 1b,d).

The surface of the carapace has numerous pores and pore‐like pits in more or less a symmetrical pattern (Figures 3, 4, 6 and 11), comprising three major types. The first type has a slit‐like opening enclosed by a conspicuous circular rim (Figures 4, 6 and 11a,c,d). These paired/symmetrical pores are most numerous and congregated in the anterior part and along the ventral and ventrolateral margins. Their number generally varies from 14 to 22 pairs. The second type is a deep pit with a round mouth from which a single short (or long in two posterior pairs) seta protrudes (Figures 4, 5 and 11a,c). Their number varies from 4 to 13 pairs. Small, paired pores (including the terminal pores of the lattice organs‐ *tp*) and bigger unpaired, so‐called central pores (*bp*), all with round mouths and possessing neither a cuticular rim nor a seta, form the third type (Figures 3b, 4, 5 and 11). All studied y‐cyprids have three unpaired central pores in the mid‐dorsal line: one anteriormost with cuticular rim and two big pores without rim representing the openings of glands (own TEM data) and associated with anterior and posterior pairs of the lattice organs. There are also 3–7 pairs of small pores without cuticular rim or seta. Unfortunately, there is no information about number and distribution of these pores in *H. acutifrons, H. furcifera, H. pacifica*, *H. rostrata*, and *H. tentaculata* and therefore this character cannot be used in taxonomical analysis. The dorsal surface of the carapace bears in the anterior half conspicuous pits or scars of muscle insertions (Figure 3a,b).

#### 3.1.1 Lattice organs (Figures 3a, 4, 5 and 11)

We found six bilateral pairs of the lattice organs (*lo 1–6*) in y‐cyprids from Kamchatka (Figures 4 and 5) and Kuril‐Kamchatka Deep Trench (Figure 11d,e), as well as in *H. itoi* (Figure 11c) and *H. spiridonovi* (Figure 11a,b). The lattice organs are situated near the mid‐dorsal line of carapace and grouped into three anterior and three posterior pairs (Figure 3a). All lattice organs are demarcated from the general cuticle by a weak depression with smooth, slightly wrinkled cuticle without tiny pores/pits comprising a pore field. The anteriormost *lo1* is smaller than other lattice organs and has a teardrop‐like form, 6.7–8.6 μm long and 2.9–3.6 μm wide (Figures 4a,b,e,f and 11c–e) or elongated, 7 μm long and 1 μm wide (Figure 11a,b), small terminal pore situates in the end opposite to the mid‐dorsal line and should be considered as posterior. The *lo1* are perpendicular to the mid‐dorsal line and located 14–20 μm from anterior margin and 15–22 μm from mid‐dorsal line. The *lo2* and *lo 3* are situated on small bumps with an anterior apical large central pore (*bp*; Figure 3a), they are elongated with wider anterior ends (Figures 4a,b,c,d and 11a,d) or teardrop‐like form (Figure 11c,d), with small posterior terminal pores. The *lo2* are 7.9–11.3 μm long and 2.0–5.0 μm, converge anteriorly and located 1.4–2.0 μm from mid‐dorsal line and 35–45 μm from anterior end. The *lo 3* lay 4.2–6.7 μm behind *lo2*, 13.2–31.7 μm long and 2.6–4.3 μm wide and located 7.7–9.1 μm from mid‐dorsal line.

The posterior pairs (*lo4‐6*) are situated close to the posterior margin of carapace, on small bump around posterior apical large central pore (*bp*), a pair of big pits with long seta inside lies laterally to the *lo6* in all studied y‐cyprids (Figures 3a and 5a,b). All posterior lattice organs with tiny posterior terminal pores (Figure 5). The *lo4* sit in front of the posterior large central pore and converge strongly anteriorly so their anterior ends almost touching (Figure 5c), slightly elongated, teardrop‐like form, 8.4–11.2 μm long and 2.2–4.1 μm wide. The *lo5* and *lo 6* lie within posteriorly tapered cuticular ridges or keels. The *lo 5* converge weakly anteriorly or almost parallel, with somewhat slightly curved posterior ends, elongated, 8.0–12.1 μm long and 1.5–2.1 μm wide, located 6.0–10.0 μm from mid‐dorsal line (Figure 5a,b,d). The *lo6* almost parallel or converge weakly posteriorly or anteriorly, elongated, 12.0–14.0 μm long and 1.5–3.0 μm wide, located 8.1–16.8 μm from mid‐dorsal line (Figure 5a,b,e).

### 3.2 Cephalic appendages (Figures 6 and 7; Table 1)

Using the terminology of Itô and Takenaka (1988), a complex of organs including the antennules, labrum, paraocular process, postocular filamentary tuft, and two pairs of rudiments of antennae and mandibles is situated under the compound eyes of y‐cypris larvae (Figures 6 and 7).

#### 3.2.1 Antennules (Figure 6)

The antennules of y‐cyprids, including described and undescribed species, seem always to consist of four segments, these being rather similar in all studied species and specimens (Figure 6a,b,f). The large, first segment may actually consist of several fused ones (Bresciani, 1965; Schram, 1970). Such fusion is also argued for the basal segment in cirripede cyprids (Høeg et al., 2003). The first segment has external cuticular folds and resembles the basal antennular segments in other thecostracan cyprids in lacking any armament (Figure 6a,b,f). The second segment is horseshoe shaped and resembles the attachment third segment of the antennule in cirripede cyprids. In many y‐cyprids, this segment (Table 1), including all from the “*Hansenocaris pacifica*”‐group, is armed with a conspicuous curved hook (“claw”) at the distal margin, putatively serving for host attachment (Figure 6). This claw is significantly larger than the second segment in y‐cypris from the Deep Kuril‐Kamchatka Trench, while it is comparable (smaller) with this segment in other y‐cyprids (Figure 6f,g). Three species (*H. acutifrons*, *H. rostrata*, and *H. tentaculata*) lack this claw altogether (Table 1). The minute lateral seta presents on the outer surface of second segment (Figure 6g). This seta was firstly described by Grygier (1987) and afterward we found it in all SEM‐examined larvae. The third, short segment bears one lanceolate seta and one small seta on the distal margin (Figure 6f,g). Such setation is characteristic for all y‐cyprids thoroughly studied with SEM (Grygier, 1987; herein). But for *H. acutifrons* is indicated “a strait spine and an aesthetasc” (Itô, 1985), one small simple seta for *H. pacifica* (Itô, 1984a), an aesthetasc and bifid short seta for *H. rostrata* (Itô, 1984b) and one spiniform seta for *H. tentaculata* (Itô, 1986). Since these species were studied with a light microscopy only, some details may have been missed or misinterpreted. The fourth segment is small and armed terminally with one long, distally serrated seta, one very short seta and one “thorn” probably representing a rudimentary seta (Figure 6f,g). Subterminally, it also carries a long aesthetasc (Figure 6). The form of aesthetasc is different: it may be narrow, ribbon shaped (Table 1; Figure 6c,d) or having bulbous proximal and distal parts separated by a very characteristic constriction (Table 1; Figure 6a,b,e–g). For *H. rostrata* and *H. acutifrons*, Itô described in the fourth segment only two setae (short and long), while a subbasal aesthetasc was indicated for the third segment, probably erroneously (Itô, 1984b, 1985).

#### 3.2.2 Labrum (Table 1; Figures 6a,b and 7b–f)

The majority of y‐cyprids possess a prominent labrum consisting of the wider basal/proximal part and a more or less bulbous distal part (Figure 7b–d). The labrum is reduced to a blunt swelling in *H. acutifrons* (Itô, 1985) or entirely absent in y‐cypris from the Deep Kuril‐Kamchatka Trench (Table 1).

The wider basal part of the labrum has a wrinkled cuticle and covers a slit with a mouth (Figure 7d). This part bears a pair of big papilliform lateral pores with a cuticular rim (Figure 7c,d). The distal part of the labrum has one anterior curved long spine and four posterior long spines or hooks (Figure 7b–f), one or two big unpaired pores with cuticular rim on posterior edge (Figure 7b–d) and proximally a pair of small lateral pores without a rim (Figure 7c). Tiny paired (1–2) and unpaired (1) pores without a rim are present at the bases of the posterior spines (Figure 7f).

#### 3.2.3 Rudiments of antennae and mandibles (Figures 6a and 7a)

It seems that all y‐cyprids retain rudiments of antennae and mandibles. Like previous authors (Grygier, 1987; Itô, 1984a, 1989; Itô & Ohtsuka, 1984), we found two pairs of small wrinkled hillocks, 13–17 μm in diameter (Figure 6a), just behind the antennules and lateral to the labrum. Sometimes the recently molted y‐cyprids of *H. itoi* possess conspicuous bifurcated rudiments, thus supporting the supposition about antennal and mandibular origin of these structures (Figure 7a).

#### 3.2.4 Paraocular process and postocular filamentary tuft (Table 1; Figures 6a,b and 7a,c,d,g)

The paraocular processes and postocular filamentary tufts are located laterally to the bases of antennules (Figures 6a and 7a). Often these structures are hidden by the margin of carapace, but they are found in all facetotectan species and also in unidentified y‐cyprids (see Table 1). Thus, their presence likely represents the ground pattern feature for all Facetotecta. The paraocular processes are bifurcated and connected by a basal part with the co‐lateral compound eye (Figure 7a). Both position and shape suggest that they represent the external portion of the organs of Bellonci (Itô & Takenaka, 1988), therefore being homologous to the frontal filaments and glendronach of other thecostracan larvae (Walker, 1974; Høeg et al., 2003; own unpublished TEM images). The paraocular process consists of a swollen basal part and narrow bifurcate distal part terminating with anterior and posterior protrusions or rami (Figures 6a,b and 7a,g). Normally these rami are equal and not strongly elongated, and the paraocular process is shorter than the antennule. Only in *H. tentaculata* the process is longer than the antennule, and with unequal length rami (Itô, 1986; Table 1).

A pair of postocular filamentary tufts is situated posteriorly to the paraocular processes (Figure 7a). Each tuft consists of a proximal cylindrical stalk that is normally hidden by the margin of carapace (Figure 7a) and a distal part with 9–15 setiform protrusions (Figures 6b and 7a,c,d). It was shown by Itô and Takenaka (1988) that these tufts have a secretory nature.

### 3.3 Thorax and thoracopods (Figures 1, 2 and 8)

The thorax consists of six segments (thoracomeres) bearing biramous appendages (thoracopods). Thoracomeres 2–6 have tergites with serrate posterior margins, and the last two thoracomeres have pleural extensions with different degree of development (Figures 1, 2 and 8a,b). The first thoracomere has no individual tergite and is distinct only ventrally (Figure 8e). Based on thoracopod 1 muscle insertions, Grygier (1987) supposed that first two thoracomeres are dorsally fused, although this supposition needs to be proved by further TEM studies as a fusion of thoracomere 1 with the head is also possible. Each tergite is also equipped with two or three transverse and several short longitudinal cuticular ridges (Figures 1 and 8a,b). In all y‐cyprids, the last two thoracomeres have pleural extensions. In *H. acutifrons*, *H. papillata*, *H. rostrata*, *H. tentaculata*, and some y‐cyprids from Kamchatka these extensions possess trapezoidal margins and sharp posterior ends (Figures 1c, 2c,f and 8a), while in *H. furcifera*, *H. itoi*, *H. pacifica*, and some other y‐cyprids from Kamchatka they are rather rounded (Figures 1a, 2d,e and 8b). The lateral articulate membrane covering thoracopod insertions bears four irregular proximal sclerites or “coxicules” (Grygier, 1987; Itô, 1989; Figure 8a—“*a, b, c, d*”). The size of these coxicules decreases from proximal to distal ones. Basal coxal sclerites are located on the ventral side (Figure 8e). The ventral sternites bear three sclerotized plates or bars (Figure 8e). The rod‐like medial plate (erroneously called “*intercoxal plate awamori*” in terminology of Itô, 1989) is inserted between the basal coxal sclerites and does not connect with the thoracopod coxae; two shorter and wider plates lie anteriorly and posteriorly.

Each thoracopod (Figure 8) consists of a basal array of sclerites, a coxa, a basis, and a pair of rami (exopod and endopod). Except *H. tentaculata*, all y‐cyprids have a more or less identical thoracopod segmentation that can only be seen at the ultrastructural level. In *H. tentaculata* thoracopods 1 and 2 have two‐segmented exopods but unsegmented endopods, while other the thoracopods possess two‐segmented endo‐ and exopods. The first thoracopod in other y‐cyprids has two‐segmented endo‐ and exopods, each with a short proximal segment lacking armament and an elongate distal segment bearing two terminal setae with rare long setules (Figure 8c,d). The distal parts of the segments bear villiform denticles of different length. The distal endopod segment may have a distinct middle constriction marked with ctenoid denticles and thus indicating a fusion of two original distal segments of the endopod (Figure 8d). The exopods of the remaining thoracopods (2–6) are two segmented as in the first pair, but they have three, instead of two, terminal setae—the outer seta is shorter than two long middle and inner setae (Figure 8c,d,f). All Kamchatka y‐cyprids as well as *H. furcifera*, *H. itoi*, and y‐cypris from Atlantic (Grygier, 1987) have three‐segmented endopods of thoracopods 2–6; the second segment has a single, long inner seta on its distal end while the distal segment bears two long terminal setae (Figure 8c–f). The protopod of thoracopod 6 is shorter than the other ones. Other y‐cyprids (*H. acutifrons*, *H. pacifica*, *H. papilata*, *H.rostrata*, *H. spiridonovi*, and y‐cypris from Deep Kuril‐Kamchatka Trench) possess two‐segmented endopods with the same setation as in three‐segmented ones, where the inner seta in the middle part marks the merging of the two distal segments. A conspicuous constriction is often present at the merging site of these two distal segments.

### 3.4 Abdomen and furcal rami (Table 1; Figures 1, 2, 9 and 10)

In all y‐cyprids, except *H. tentaculata*, the abdomen consists of three short segments and a long telson with two furcal rami (Figures 1, 2 and 9). In *H. tentaculata*, the abdomen encompasses only a single short segment and a long telson (Itô, 1986). The abdominal segments are sculptured with transverse and longitudinal cuticular ridges and serrate margins (Figure 9a,b,d,f). Each tergite of the abdominal segments often has sharp and long lateral (pleural) extensions (Table 1; Figure 9a,b,d). These pleural extensions are longer in the second and third somites and may be shorter (Figure 9a) or reduced (Figure 1d) in the first. The pleural extensions are reduced in all abdominal segments in *H. papillata* (Figure 1c), *H. acutifrons*, *H. pacifica*, *H rostrata*, and *H. tentaculata* (see Table 1). Tergites of abdominal segments may bear pores/pits with or without seta inside (Figure 9b), while the ventral surfaces have a pair of small, rounded pores at the bases of pleural extensions (Figure 9c). The midventral part of the first abdominal segment has a small and bifurcated outgrowth (Figures 8f and 9c) interpreted as a putative penis rudiment (Grygier, 1987; Itô, 1989).

The telson is densely covered by chitinous serrate cuticular ridges, forming dorsal, lateral, and ventral longitudinal rows of plates, having normally more or less rectangular shape and symmetrical pattern that is sometimes broken towards the posterior end (Figures 1, 2 and 9a–f). In the majority of y‐cyprids, the telson has two dorsal rows of cuticular plates; each lateral side bears two rows, laterodorsal and lateroventral; the ventral surface ordinary consists of five indistinct, longitudinal rows of plates (one central, two ventrolateral, and two ventromedial). The telson in *H. tentaculata* has only two irregular rows of dorsal plates, one row of elongated lateral plates, and no distinct plates on ventral side (Itô, 1986). The surface of the telson bears several conspicuous papilliform pores (Figure 9g) distributed on dorsal (1–2 pairs), lateral (2–3 pairs), and ventral (1–2 pairs) sides. A pair of pits with a short seta inside sits at the posteriormost end of telson outside to the bases of furcal rami (Figure 10c,d). The number of cuticular plates in each row and the number and position of papilliform pores vary within species and thus have a taxonomical value in Facetotecta. The posterioventral border of the telson is armed with 4–6 (ordinary 5) conspicuous and serrate terminal spines also having a taxonomical value (number) but is absent in *H. tentaculata* (Figures 9a,c and 10a,b; Table 1).

A pair of short furcal rami is inserted at the posterior end of the telson (Figures 1a, 9a,b,e and 10). Each ramus resembles a two‐annulated structure due to the more or less developed circular cuticular ridge (Table 1; Figure 10c,d), but it is in fact unsegmented (unjoined). The furcal rami of almost all described species and studied y‐cyprids carry three wide, lanceolate setae of different lengths, with serrate margins (Figure 10b, *nos 2–4*, Table 1), but *H. acutifrons* differs in possessing only two such setae (Itô, 1985). In all y‐cyprids studied with SEM, we found a tiny distal seta with terminal pore (*no 1*) and an outer basal papilla with pore (Figure 10b–d).

## 4 DISCUSSION

### 4.1 Morphological similarities and differences between y‐cyprids of Facetotecta

The diversity of the Facetotecta is seen best in the y‐cypris. Ultrastructural examinations of y‐cypris larvae are crucial to understanding the structural and biological diversity of the Facetotecta, not least because this stage is putatively involved in host location and attachment. This stage possesses a suite of morphological characters that are useful for species delimitation. This calls for a systematic investigation and assessment of y‐cypris characters useful for future systematic studies on y‐larvae.

For this purpose, it is necessary to first determine the characters common and unique to all y‐cyprids and separating them from other thecostracan cypridiform larvae. Primarily these are (i) univalved carapace having an inverted boat form and covering the body only dorsally; (ii) a long telson with well‐developed cuticular ridges forming cuticular plates; (iii) presence of only five thoracic tergites; (iv) pleural extensions on last two thoracomeres; (v) unique four‐segmented antennules; and (vi) special cephalic appendages—bifurcate paraocular process and postocular filamentary tuft and (vii) six pairs of the lattice organs. While ascothoracid (cypridiform) larvae have a bivalved carapace, cirripede cyprids possess univalved carapace (i.e., without a clear dorsal hinge structure), but in both taxa it covers the whole larval body from dorsal, lateral, and ventral sides. The carapace most of y‐cyprids is ornamented with conspicuous cuticular ridges forming cuticular plates at least in the anterior part. But several cypridiform larvae of Ascothoracida and Cirripedia also possess cuticular ridges or grooves of carapace (as well as perforations) forming honey comb (ascothoracid larvae of *Baccalaureus* or cyprids of *Cryptophialus*, *Lepas*, and *Pollicipes*) or reticulated (ascothoracid larvae of *Dendrogaster*) patterns (Kolbasov et al., 2008; Kolbasov, 2009; own data). The presence of such ornamentations seems not to be correlated with cypris size and their putative adaptational function is completely obscure. While cirripede cyprids have a highly reduced abdomen with short telson (Kolbasov et al., 1999), the ascothoracid larvae possess developed 4–5‐segmented abdomen with long telson (Kolbasov et al., 2008; own data), although their telson lacks cuticular ridges forming plates, is flattened laterally and bears only a pair of telsonic spines (in contrast to normally 4–6 telsonic spines in y‐cyprids, see Table 1). All six thoracomeres in cypridiform larvae of Ascothoracida and Cirripedia are separated but the tergite of first thoracomere in y‐cyprids is fused with the cephalon or with the second thoracomere in Grygier's (1987) interpretation. The well‐developed pleural extensions of the last two thoracomeres are characteristic for y‐cyprids and absent in other thecostracan cypridiform larvae. The antennules of the generalized (i.e., putative plesiomorphic) cypridiform larva of the Ascothoracida are six segmented, although in those of *Dendrogaster* are apomorphic in having four‐segmented antennules, as in y‐cyprids. But a clear difference is that in Ascothoracida the claw always sits on distal antennular segment, while it sits on the second in Facetotecta. The cirripede cyprids also possess four‐segmented antennules, but they lack a curved hook comparable to that in y‐cyprids, and their third segment bears an attachment disc covered with cuticilar villi and having exit pores for both the multicellular cement gland and unicellular glands (Bielecki et al., 2009; Høeg et al., 2003). Thus four‐segmented antennules of Facetotecta are unique and not homologous to four‐segmented antennules of other thecostracan cypridiform larvae (see also Grygier, 1987). On the other hand, it is very likely that the fourth segment of cirripede cyprids evolved by fusion of the two distal segments in y‐cyprids. Itô and Takenaka (1988) established that the paraocular process is connected with the compound eye and thus putatively homologous to the frontal filaments of other thecostracan larvae, but its bifurcate shape is unique within Thecostraca. The postocular filamentary tufts are characteristic only for y‐cyprids and absent in other thecostracans. They may therefore represent an autapomorphy for the taxon.

#### 4.1.1 Lattice organs

Although the previous characters were already known for y‐cyprids, the presence of six instead five pairs of the lattice organs is described here for the first time. The presence of five (two anterior and three posterior) pairs of the lattice organs was seen as a fundamental symplesiomorphic character of the carapace for all Thecostraca (Høeg & Kolbasov, 2002; Jensen et al., 1994). The lattice organs were subsequently described in Cirripedia, Ascothoracida, and Facetotecta (Elfimov, 1986; Høeg & Kolbasov, 2002; Itô & Grygier, 1990; Jensen et al., 1994). The lattice organs have a similar anatomy throughout and are homologous in all Thecostraca. TEM reveals that they are chemoreceptors and evolved originally from free setae (Høeg et al., 1998). The external morphology and anatomy of the lattice organs may also indicate their homology with dorsal organs (anterior and posterior) found in the carapace of Malacostraca (Lerosey‐Aubril & Meyer, 2013). Both represent chemosensory structures grouped around unpaired anterior and posterior glands. Unfortunately, innervation (from the tritocerebrum) was established only for anterior dorsal organ in one species of Anaspidacea (Hanstrøm, 1947) and one species of Decapoda (Laverack & Sinclair, 1994), and there is no information about the innervation of posterior dorsal organ and the lattice organs.

Høeg and Kolbasov (2002) described 5 pairs of the lattice organs in Facetotecta, but an extra anteriolateral pair of the lattice organs (*lo1*, see description) was here revealed in all y‐cyprids after our detailed examination with SEM. This anteriolateral pair is absent in Cirripedia and Ascothoracida (own data). The number (5) of the lattice organs may evidence on their segmentary nature because five segments (a ground pattern for Crustacea) are incorporated in the head of Ascothoracida and Cirripedia being covered by carapace. If the first thoracomere fuses with cephalon forming cephalothorax (5 + 1), instead with second thoracomere, as Grygier (1987) suggested, this could explain the presence of six pairs of the lattice organs in y‐cyprids. In parasitic Tantulocarida, which may belong to Thecostraca (see Petrunina et al., 2014), the free‐swimming males possess a carapace resembling that in y‐cyprids of Facetotecta (Petrunina & Kolbasov, 2012). This carapace bears seven pairs of big pores/pits with a tuft of sensillae inside that are likely homologous to the lattice organs, although this is pending ultrastructural investigation. Interestingly, the tantulocaridan males also possess a cephalothorax that incorporates the two first thoracomeres (5 + 2). This may explain the presence of seven pairs of sensillate pores. Rybakov et al. (2003) showed that the lattice organs in cyprids correspond to setae in the preceding nauplius, thus ontogeny also emphasizes the setal origin of these fascinating structures in thecostracan cypridiform larvae.

Obviously, the anatomy and innervation of these chemosensory structures must be studied to establish possible homologies within Thecostraca, Tantulocarida and Malacostraca.

In spite of a number of common characters shared by y‐cyprids of Facetotecta, they differ between species and forms in many other respects (see Results and Tables 1 and 2). These are (i) shape, size, and armament of carapace; (ii) presence or absence and size of hooks of antennules; (iii) form of antennular aesthetasc; (iv) development of labrum; (v) size of rami in paraocular process; (vi) segmentation of thoracopods; (vii) segmentation, armament, and ornamentation of abdomen; and (viii) setation of furcal rami.

**TABLE 2:** Character matrix. Unknown states marked by (?), inapplicable by (−)

Characters	1	2	3	4	5	6	7	8	9	10	11	12	13	14	15
Species or specimens															
*H. acutifrons*	1	1	1	1	0	1	–	0	1	0	0	0	1	0	1
*H. furcifera*	0	0	0	0	0	0	0	0	0	0	0	0	0	0	0
*H. itoi*	0	0	0	0	0	0	0	1	0	0	0	0	0	0	0
*H. pacifica*	0	0	0	0	0	0	0	0	0	0	0	0	1	0	0
*H. papillata*	1	0	0	0	0	0	0	0	0	?	0	0	1	0	0
*H. rostrata*	1	1	0	1	0	1	–	0	0	0	0	0	1	0	0
*H. spiridonovi*	0	0	0	0	0	0	0	0	0	?	0	0	0	0	0
*H. tentaculata*	1	0	1	1	0	1	–	0	0	1	1	1	1	1	0
Kamchatka1	0	0	0	0	0	0	0	1	0	0	0	0	0	0	0
Kamchatka2	0	0	0	0	0	0	0	1	0	0	0	0	0	0	0
Kamchatka3	0	0	0	0	0	0	0	0	0	?	0	0	0	0	0
Kamchatka4	0	0	0	0	0	0	0	1	0	0	0	0	0	0	0
Kamchatka5	0	0	0	0	0	0	0	1	0	0	0	0	0	0	0
Kamchatka6	0	0	0	0	0	0	0	0	0	0	0	0	0	0	0
Kuril Trench	0	0	0	0	1	0	1	0	1	?	0	0	0	0	0

### 4.2 Phylogeny

Although several groups of y‐cyprids were proposed on the basis of their morphology (see Introduction here; Kolbasov & Høeg, 2003; Kolbasov et al., 2008), there is no cladistic analysis to formalize the relations between described facetotectan species and unspecified y‐cyprids. As a pioneering attempt, we here use the characters of y‐cyprids to align them cladistically and determine the limits of the genus *Hansenocaris* s.s. We are perfectly aware that the optimum pathway is a joint analysis by both molecular and morphological means. But unfortunately, the molecular sequences are absent for most of the species of Facetotecta, and the molecular phylogeny in Pérez‐Losada et al. (2009) does not incorporate comprehensive and specimen‐based character resolution for their y‐cyprids. Therefore, we conduct a morphological analysis and argue that the character matrix here set up will be of much future value, even if the topology of the tree will undoubtedly see future changes.

For all described species of Facetotecta and SEM studied y‐cyprids we developed a matrix of 15 characters for the Nexus Data Editor 5.0 (Table 2). Data were scored “0” or “1,” when both conditions were present (we avoided multistate data), “‐” for inapplicable states, and “?” for unknown state. Eight characters (numbers 1, 2, 3, 4, 6, 8, 9, 13) are parsimony informative. Uninformative characters do not contribute to this parsimony analysis. But they rather represent synapomorphies for y‐cyprids and, therefore, we consider these features as very important in the understanding facetotectan evolution.

*List of characters*

1. Size of carapace: 0 = posteriolateral corners of carapace reaching 5th thoracomere or more; 1 = posteriolateral corners of carapace reaching 4th thoracomere or less.
2. Length of anterior end of carapace: 0 = anterior end not strongly produced; 1 = anterior end strongly produced.
3. Form of anterior end of carapace: 0 = rounded; 1 = sharp.
4. Cuticular ridges of carapace: 0 = present at least in anterior part; 1 = reduced (“very faint”) in whole surface.
5. Exterior of carapace: 0 = not perforated; 1 = perforated.
6. Hooks of antennules: 0 = present; 1 = absent.
7. Size of hook of antennules (if applicable): 0 = comparable (smaller) with second segment; 1 = significantly larger than second segment.
8. Form of antennular aesthetasc: 0 = narrow, ribbon shaped; 1 = bulbous, with middle constriction.
9. Development of labrum: 0 = well‐developed, cone‐shaped; 1 = reduced or absent.
10. Rami of paraocular process: 0 = equal, shorter than a1; 1 = unequal, one ramus longer than a1.
11. Endopod of thoracopod 1: 0 = two‐segmented; 1 = unsegmented.
12. Number of abdominal segments (exclude telson): 0 = 3 segments; 1 = 1 segment.
13. Pleural extensions of abdominal segments: 0 = developed, sharp; 1 = reduced.
14. Serrate spines along the posterioventral margin of the telson: 0 = present; 1 = absent.
15. Number of lanceolate setae of furcal ramus: 0 = 3 setae; 1 = 2 setae.

**FIGURE 12:**
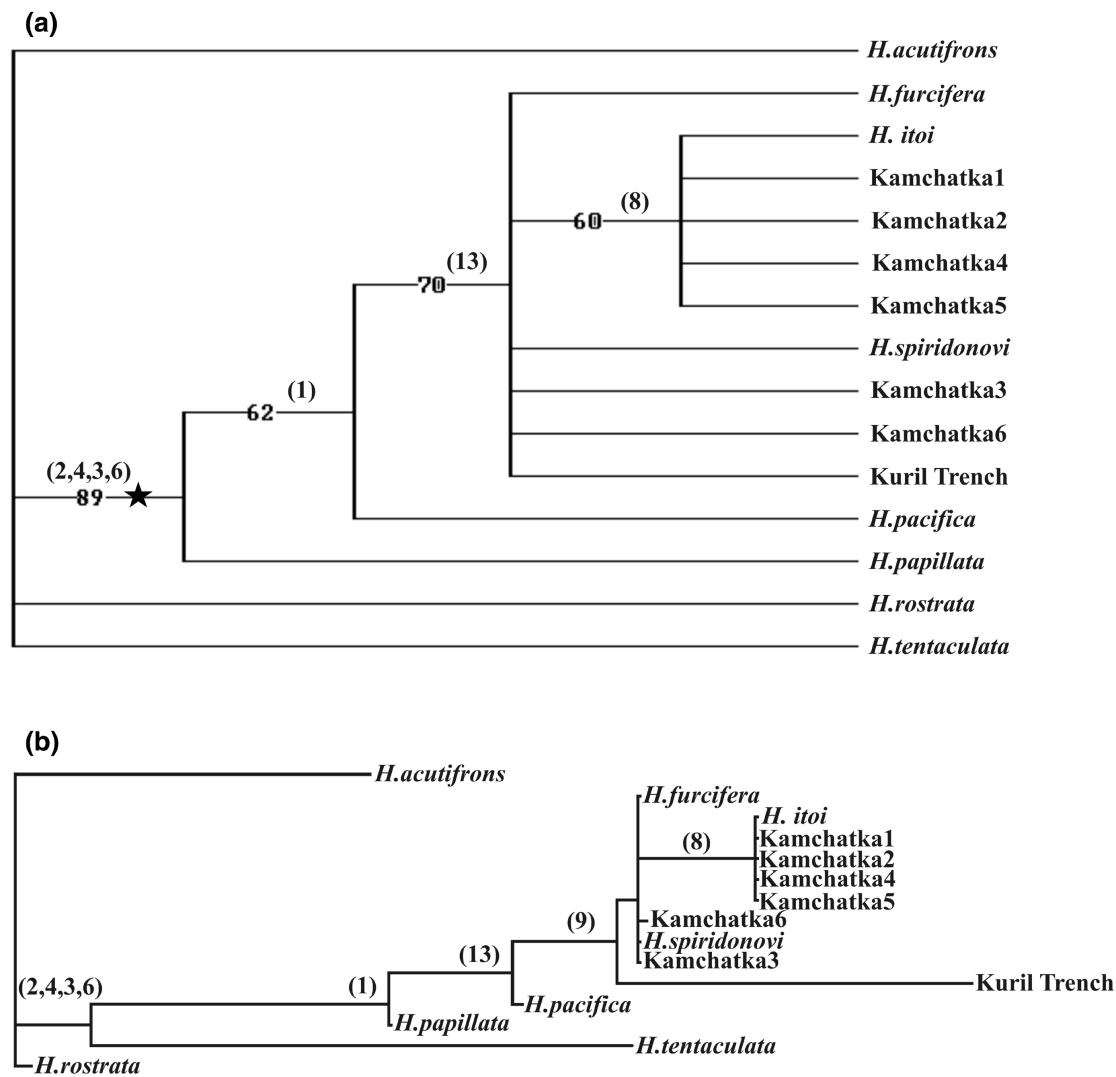
Cladistic reconstruction cladogram of the species and y‐cyprids of Facetotecta (all characters unordered and of equal weight; PAUP, Swofford, 1998; parsimony‐informative characters indicated in brackets): (a) Bootstrap 50% majority‐rule consensus tree (node corresponding to “*H. pacifica* ‐ group” indicated by star). Percentages at nodes denote frequency of occurrence among 100 trees. (b) Neighbor‐joining tree.

These data were subjected to parsimony analysis and a search of the shortest trees (PAUP 4.0, Swofford, 1998). All characters were entered unordered and of equal weight, and all trees were unrooted. We reconstructed bootstrap 50% majority‐rule consensus and neighbor‐joining trees (Figure 12). These trees show that species *H. itoi*, *H. furcifera*, *H. pacifica*, *H. papillata*, and *H. spiridonovi* form a monophyletic clade with y‐cyprids from Kamchatka and Kuril‐Kamchatka Deep Trench. This clade corresponds to the “*Hansenocaris pacifica*” group. But y‐cypris from Kuril‐Kamchatka Deep Trench has a rudimentary labrum and an antennular hook significantly larger than second segment, and we argue that this delimits the form from the genus *Hansenocaris* s.s. Thus, the genus *Hansenocaris* s.s. is characterized by (i) more or less elongated carapace with rounded anterior end and developed cuticular ridges at least at anterior part; (ii) developed labrum with five long spines; (iii) antennules with hook smaller or comparable with second segment; (iv) paraocular process with equal rami and shorter than antennules; (v) abdomen four‐segmented; (vi) telson with serrate spines along posterioventral margin.

Other species, such as *H. acutifrons*, *H. rostrata* and *H. tentaculata* should be excluded from the genus *Hansenocaris* s.s. in having shorten carapace with reduced cuticular and elongated, sharp anterior end and absence of the antennular hook. Besides *H. tentaculata* possessing only two‐segmented abdomen and enlarged paraocular process and is unique within Facetotecta. We suppose that each of these species forms separate taxa (genera at least). On the other hand, we presently abstain from formal taxonomic steps. This is best done on a much larger collection of species, where at least a considerable number are also characterized by molecular markers.

### 4.3 Summary and outlook

One of the most provoking results of our study is the presence in y‐cyprids of six instead five pairs of the lattice organs. The presence of five (two anterior and three posterior) pairs of the lattice organs having a similar anatomy was seen as a synapomorphy and a fundamental character of the carapace for all Thecostraca. Høeg and Kolbasov (2002) described five pairs of the lattice organs in Facetotecta, but an extra anteriolateral pair of the lattice organs was here revealed in all y‐cyprids. This anteriolateral pair is absent in Cirripedia and Ascothoracida. The number of the lattice organs may evidence on their segmentary nature in Thecostraca.

The y‐cyprids share a number of common and unique characters: (i) relatively short univalved carapace having an inverted boat form; (ii) a long telson with well‐developed cuticular ridges forming cuticular plates; (iii) presence of only five thoracic tergites; (iv) pleural extensions on last two thoracomeres; (v) unique four‐segmented antennules; (vi) special cephalic appendages—bifurcate paraocular process and postocular filamentary tuft and (vii) six pairs of the lattice organs. But in spite of a number of common characters, y‐cyprids differ in many other respects: (i) shape, size, and armament of carapace; (ii) presence or absence and size of hooks of antennules; (iii) form of antennular aesthetasc; (iv) development of labrum; (v) size of rami in paraocular process; (vi) segmentation of thoracopods; (vii) segmentation, armament and ornamentation of abdomen and (viii) setation of furcal rami. These differences evidence on the presence of several separate genera instead of a single *Hansenocaris*. The characters reviewed and discussed here will be useful for future phylogenetic efforts, in which species are grouped on both molecular and morphological characters.

The genus *Hansenocaris* s.s. is characterized by (i) more or less elongated carapace with rounded anterior end and developed cuticular ridges at least at anterior part; (ii) developed labrum with five long spines; (iii) antennules with hook smaller or comparable with second segment; (iv) paraocular process with equal rami and shorter than antennules; and (v) abdomen four segmented; (vi) telson with serrate spines along posterioventral margin.

## AUTHOR CONTRIBUTIONS

**Gregory A. Kolbasov:** Conceptualization (equal); data curation (equal); formal analysis (equal); funding acquisition (equal); investigation (equal); supervision (equal); writing – original draft (equal). **Alexandra S. Savchenko:** Conceptualization (equal); data curation (equal); formal analysis (equal); investigation (equal); writing – original draft (equal). **Niklas Dreyer:** Methodology (equal); writing – review and editing (equal). **Benny K. K. Chan:** Conceptualization (equal); data curation (equal); funding acquisition (equal); investigation (equal); writing – original draft (equal). **Jens T. Høeg:** Conceptualization (equal); data curation (equal); investigation (equal); methodology (equal); writing – original draft (equal).

## FUNDING INFORMATION

For GAK and ASS, this work was financially supported by the Russian Foundation for Basic Research (grant 21‐54‐52003 MNT_a). BKKC is supported by the Taiwan Russia bilateral grants from Ministry of Science and Technology, Taiwan (MOST‐110‐2923‐B‐001‐003‐MY3). ND is jointly supported by a double‐degree graduate grant from the Biodiversity Research Center, Academia Sinica, Taiwan and the Natural History Museum of Denmark (University of Copenhagen).

## CONFLICT OF INTEREST

The authors declare no competing interests.

## Data Availability

All morphological data will be upload to Dryrad upon acceptance. SEM stubs are stored in the Kolbasov lab at the Moscow State University and can provide for examination upon on request.
